# Potent Tetrahydroquinolone Eliminates Apicomplexan Parasites

**DOI:** 10.3389/fcimb.2020.00203

**Published:** 2020-06-09

**Authors:** Martin J. McPhillie, Ying Zhou, Mark R. Hickman, James A. Gordon, Christopher R. Weber, Qigui Li, Patty J. Lee, Kangsa Amporndanai, Rachel M. Johnson, Heather Darby, Stuart Woods, Zhu-hong Li, Richard S. Priestley, Kurt D. Ristroph, Scott B. Biering, Kamal El Bissati, Seungmin Hwang, Farida Esaa Hakim, Sarah M. Dovgin, Joseph D. Lykins, Lucy Roberts, Kerrie Hargrave, Hua Cong, Anthony P. Sinai, Stephen P. Muench, Jitender P. Dubey, Robert K. Prud'homme, Hernan A. Lorenzi, Giancarlo A. Biagini, Silvia N. Moreno, Craig W. Roberts, Svetlana V. Antonyuk, Colin W. G. Fishwick, Rima McLeod

**Affiliations:** ^1^School of Chemistry, The University of Leeds, Leeds, United Kingdom; ^2^Department of Ophthalmology and Visual Sciences, The University of Chicago, Chicago, IL, United States; ^3^Experimental Therapeutics Branch, Walter Reed Army Institute of Research, Silver Spring, MD, United States; ^4^Department of Pathology, The University of Chicago, Chicago, IL, United States; ^5^Department of Biochemistry and Systems Biology, Faculty of Health and Life Sciences, Institute of Systems, Molecular and Integrative Biology, The University of Liverpool, Liverpool, United Kingdom; ^6^School of Biomedical Sciences, Faculty of Biological Sciences, and Astbury Centre for Structural Molecular Biology, The University of Leeds, Leeds, United Kingdom; ^7^Strathclyde Institute of Pharmacy and Biomedical Sciences, The University of Strathclyde, Glasgow, United Kingdom; ^8^Department of Cellular Biology, Center for Tropical and Emerging Global Diseases, University of Georgia, Athens, GA, United States; ^9^Department of Tropical Disease Biology, Research Center for Drugs and Diagnostics, The Liverpool School of Tropical Medicine, Liverpool, United Kingdom; ^10^Department of Chemical and Biological Engineering, Princeton University, Princeton, NJ, United States; ^11^Microbiology, Immunology and Molecular Genetics, The University of Kentucky College of Medicine, Lexington, KY, United States; ^12^Animal Parasitic Diseases Laboratory (APDL), USDA-ARS, Beltsville, MD, United States; ^13^Department of Infectious Diseases, J Craig Venter Institute, Rockville, MD, United States; ^14^Department of Pediatrics (Infectious Diseases), Institute of Genomics, Genetics, and Systems Biology, Global Health Center, Toxoplasmosis Center, CHeSS, The College, University of Chicago, Chicago, IL, United States

**Keywords:** *Toxoplasma gondii*, *Plasmodium falciparum*, cytochrome bc1, tetrahydroquinolone, nanoformulation, structure-guided design, transcriptomics, RPS13Δ

## Abstract

Apicomplexan infections cause substantial morbidity and mortality, worldwide. New, improved therapies are needed. Herein, we create a next generation anti-apicomplexan lead compound, JAG21, a tetrahydroquinolone, with increased sp3-character to improve parasite selectivity. Relative to other cytochrome *b* inhibitors, JAG21 has improved solubility and ADMET properties, without need for pro-drug. JAG21 significantly reduces *Toxoplasma gondii* tachyzoites and encysted bradyzoites *in vitro*, and in primary and established chronic murine infections. Moreover, JAG21 treatment leads to 100% survival. Further, JAG21 is efficacious against drug-resistant *Plasmodium falciparum in vitro*. Causal prophylaxis and radical cure are achieved after *P. berghei* sporozoite infection with oral administration of a single dose (2.5 mg/kg) or 3 days treatment at reduced dose (0.625 mg/kg/day), eliminating parasitemia, and leading to 100% survival. Enzymatic, binding, and co-crystallography/pharmacophore studies demonstrate selectivity for apicomplexan relative to mammalian enzymes. JAG21 has significant promise as a pre-clinical candidate for prevention, treatment, and cure of toxoplasmosis and malaria.

## Introduction

Malaria results in the death of ~0.5 million children a year, with drug resistance impacting the usefulness of successive generations of new medicines (www.who.int/malaria/publications/world-malaria-report-2017/en/). The related apicomplexan parasite, *Toxoplasma gondii*, is the most frequent parasitic infection of humans in the world. It plays a significant role in food-borne associated death in the USA, destruction of the human retina (Phan et al., 2008), and death and illness from recrudescent disease in the immune compromised or immunologically immature (McLeod et al., 2006; McLeod and Boyer, 2019). It has been estimated that there are 1.9 million new cases of this congenital *T. gondii* infection globally over a 10 year period, causing 12 million disability adjusted life years (Torgerson and Mastroiacovo, 2013) from damage to the fetal brain and eye. Toxoplasmosis is an often neglected, untreated, or mistreated disease. There are ~2 billion people throughout the world who have this parasite in their brain lifelong, some with known, severe, adverse consequences (Delair et al., 2011; Wallon et al., 2013; Lykins et al., 2016). There are possible additional, harmful effects for a substantial number of chronically infected people as this parasite modulates signature pathways of neurodegeneration, motor diseases, epilepsy, and malignancies (Ngô et al., 2017). No medicine eliminates this chronic, encysted form of the parasite. New and improved medicines are greatly needed to cure *Toxoplasma* and Plasmodia infections (McLeod et al., 2006). These parasites often share the same molecular targets for medicines due to a relatively close, apicomplexan, phylogenetic relationship (McPhillie et al., 2016). Thus, medicine development for each of these parasites can inform development of medicines that benefit treating the other (Muench et al., 2007; Fomovska et al., 2012a).

One such shared molecular target is the mitochondrial cytochrome *bc1* complex that is important for the survival of apicomplexan parasites such as Plasmodia and *T. gondii*. Cytochrome *b* is a subunit of the cytochrome *bc*_1_ complex, an inner mitochondrial membrane protein that is part of the electron transport chain. Activity of this complex is integral to oxidative phosphorylation and generation of ATP (Vercesi et al., 1998). Cytochrome *b* activity appears to be necessary for the replication and persistence of the parasite (McPhillie et al., 2016), and is the site of action of atovaquone (McPhillie et al., 2016). Cytochrome *b* is the target for quinolone-based compounds, but, significant problems with solubility, and toxicity have been noted with earlier cytochrome *b* inhibitors. In an attempt to design novel quinolone-like inhibitors with improved solubility, and lower toxicity, compared to known compounds in the literature, we synthesized a series of tetrahydroquinolinones (THQs). Our preliminary efforts were described in McPhillie et al. (2016). We reasoned that the increased “sp3” character of the THQs (i.e., moving from rod-like to sphere-like 3D space) could provide the required improvement in solubility that would allow for optimal pharmacokinetic properties. Molecules with an increased percentage of “sp3 character” tend to be more three-dimensional, than their planar (“sp2-rich”) counterparts. The terms “sp2” and “sp3” refer to the shape of their hybridized atomic orbitals, which have trigonal planar and tetrahedral geometries, respectively. Flat aromatic rings (“sp2-rich”) are ubiquitous in drug discovery campaigns, but molecules with more “sp3 character” are often more specific for their protein target and can have better physicochemical properties. Further, we reasoned that the larger binding pocket in the parasite enzymes (McPhillie et al., 2016), compared to their mammalian counterparts, would provide room for bulkier substituents to minimize effect on the human enzyme. Within this new series of compounds, we aimed to identify a mature lead compound with both anti-*Plasmodium* and anti-*T. gondii* activity.

Our work developed as follows: We recently found markedly increased expression of cytochrome *b* in the currently untreatable *T. gondii* bradyzoite life-cycle stage (McPhillie et al., 2016). Thus, we set out to develop a compound that would inhibit tachyzoites, bradyzoites, and three life cycle stages of even drug-resistant Plasmodia. We sought to do this without a need for a pro-drug as has been needed in other attempts to target apicomplexan cytochrome *b* (Frueh et al., 2017). Our aim was to improve upon the physicochemical properties of napthoquinones and endochin-like quinolones (ELQs) targeting cytochrome *b*, including poor aqueous solubility and toxicity (Khan et al., 1998; Doggett et al., 2012; Capper et al., 2015; Miley et al., 2015; McPhillie et al., 2016). The intent was further to provide potential solutions for limitations of other compounds active against apicomplexan parasites (Waxman and Herbert, 1969; Caumes et al., 1995). Our concurrent crystallographic studies also enable better understanding of the interactions between ligand and the binding pocket of the Q_i_ site (McPhillie et al., 2016).

Herein, we have identified a preclinical lead candidate based on potent and selective inhibition of *Plasmodium falciparum, Plasmodium berghei*, and *T. gondii* cytochrome *bc1* for the treatment of malaria and toxoplasmosis. The candidate compound demonstrates high efficacy in relevant *in vitro* and *in vivo* models of the diseases, and has considerable potential for broad-spectrum use (i.e., against *T. gondii* tachyzoites and encysted bradyzoites, and drug resistant Plasmodia). The data which follow present the creation and characterization of this novel, broad-spectrum, anti-apicomplexan lead compound which has promise for definitive treatment of these infections.

## Materials and Methods

### Syntheses of Compounds

#### Synthesis of Tetrahydroquinolones (THQs) Compounds

**Figure 1:**
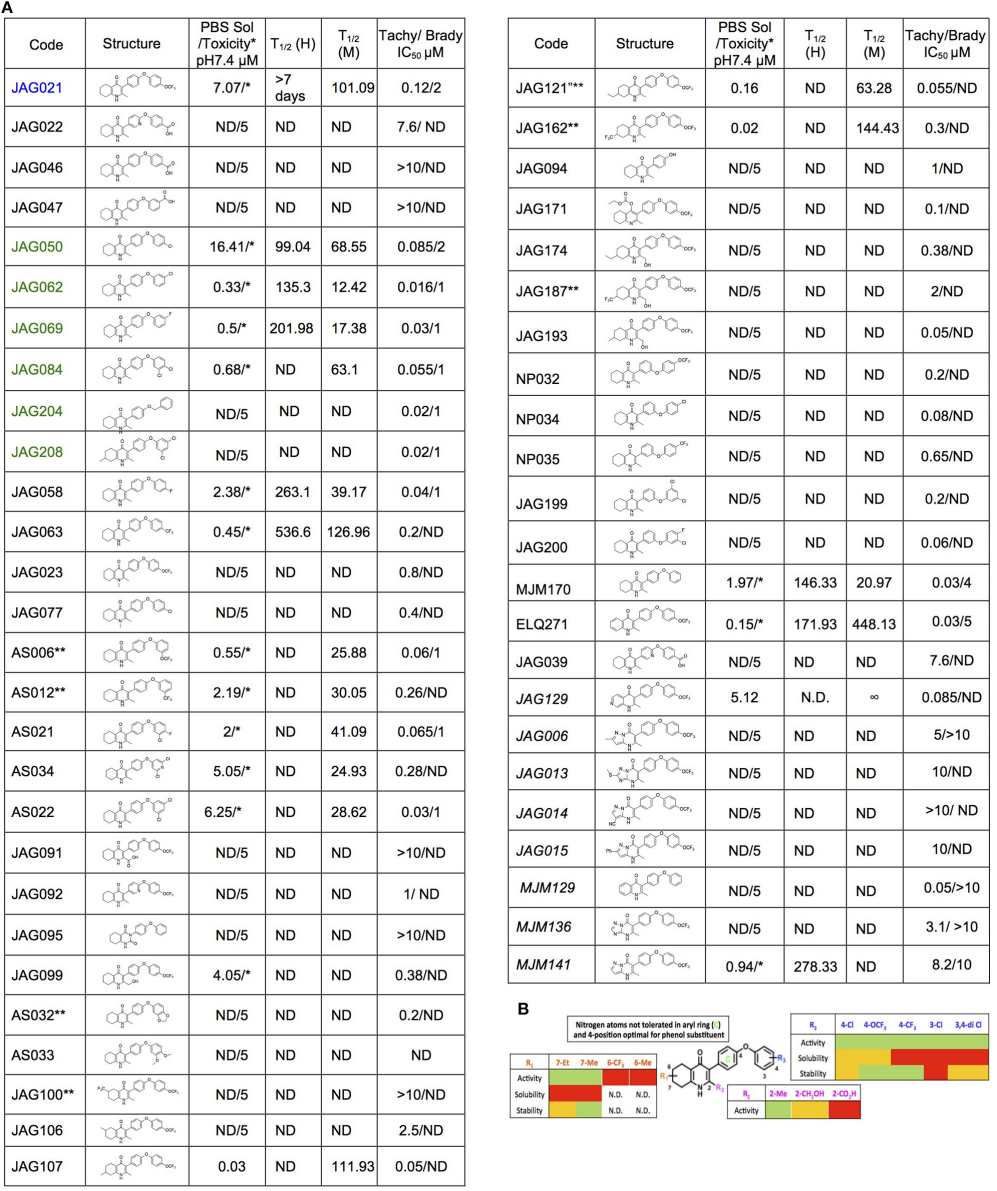
Characteristics and effects of compounds on inhibition of *Toxoplasma gondii* replication and enzyme activity, and Structure Activity Relationship analysis. **(A)** Cytochrome b/c inhibitor code, Chem Draw structure, solubility in PBS 7.4, toxicity against HFF, predicted half-life, and inhibitory effect of compounds on RH strain tachyzoites and EGS strain bradyzoites *in vitro* and saffarine O assay enzyme activity. PBS Sol/Toxicity pH7.4 refers to solubility of the compound in Phosphate Bufferred Saline (PBS) at pH 7.4. Toxicity refers to the highest concentration tested that does not show toxicity to Human Foreskin Fibroblast (HFF) in tissue culture in WST assay; T1/2 (H) refers to the predicted half-life in human liver microsomes; T1/2 (M) refers to the predicted half-life in mouse liver microsomes. Tachy/Brady IC50 was determined in studies in which cultures of parasites in HFF were treated with varying concentrations of the compound and there was 50% inhibition of the replication (number) of parasites. Parasites were RH-YFP expressing tachyzoites (Tachy) and EGS (Brady) strains. Studies of effects of inhibitors on HFF or on *T. gondii* tachyzoites were performed with triplicate wells in at least 2 biological replicate experiments. Studies of effects on bradyzoites were performed at least twice in at least 2 biological replicate experiments. Compounds with much less inhibition of mammalian than *T. gondii* cytochrome *bc*, relative to JAG21 effect on parasite enzyme, in the saffarine enzyme assay (indicated by **) provide potential to further develop compounds, if unanticipated toxicity occurs from JAG21. **(B)** Structure Activity Relationship analysis (SAR). The effects of changing R1 as 7-Et, 7-Me, 6-CF_3_, or 6-Me on activity against *T. gondii* RH strain tachyzoites, solubility, and stability were compared in the SAR. Color Key in **(B)** *Activity*: Green <50 nM, Red > 1 μM; *Solubility* in 100 mM Phosphate Buffer (pH 7.4): Amber>10 μM, Red <10 μM; *Metabolic Stability*: Green >120 min, Amber 60–120 min, red <60 min. SAR panel displays only representative structures and trends within the JAG compound series. JAG21 (blue font) is highly active, has the longest predicted half-life for humans of initial compounds tested (green), combined with improved solubility, no hERG liability, and predicted capacity to cross the blood brain barrier (BBB). Definitions of ADMET terminology are in the Materials and Methods. In summary, in the SAR overall, nitrogen atoms were not tolerated in aryl ring marked by green c, and the 4-position was optimal for phenol substituent. Compound name with “0” or no “0” between letters and number, e.g., JAG21 or JAG021, refer to the same compound. This is throughout the manuscript.

**Scheme 1:**
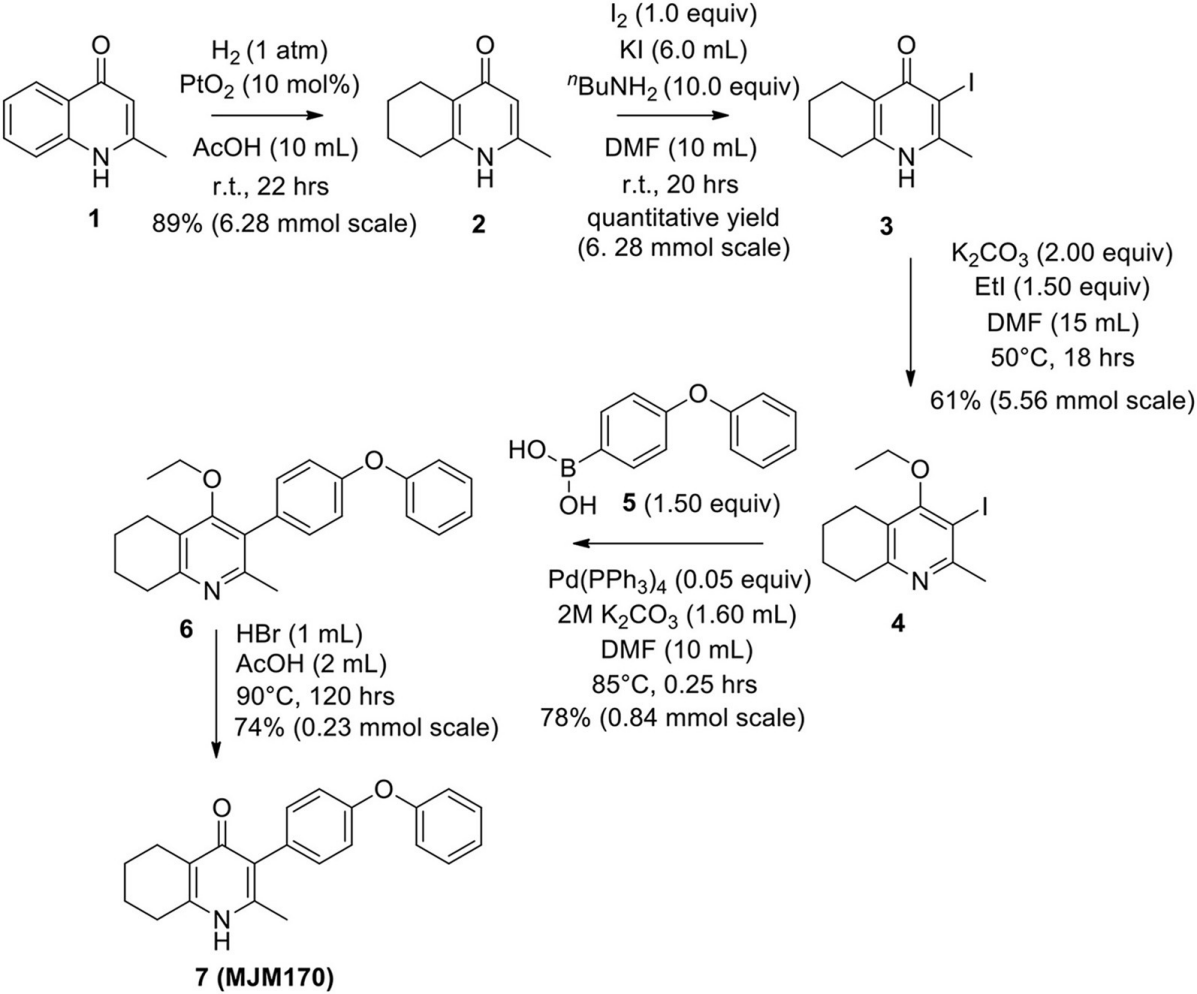
Synthesis of hit compound 7, also known as MJM170 (McPhillie et al., 2016). Synthetic scheme inspired by the route to endochin-like quinolones (ELQs) reported by Doggett et al. (2012).

**Scheme 2:**
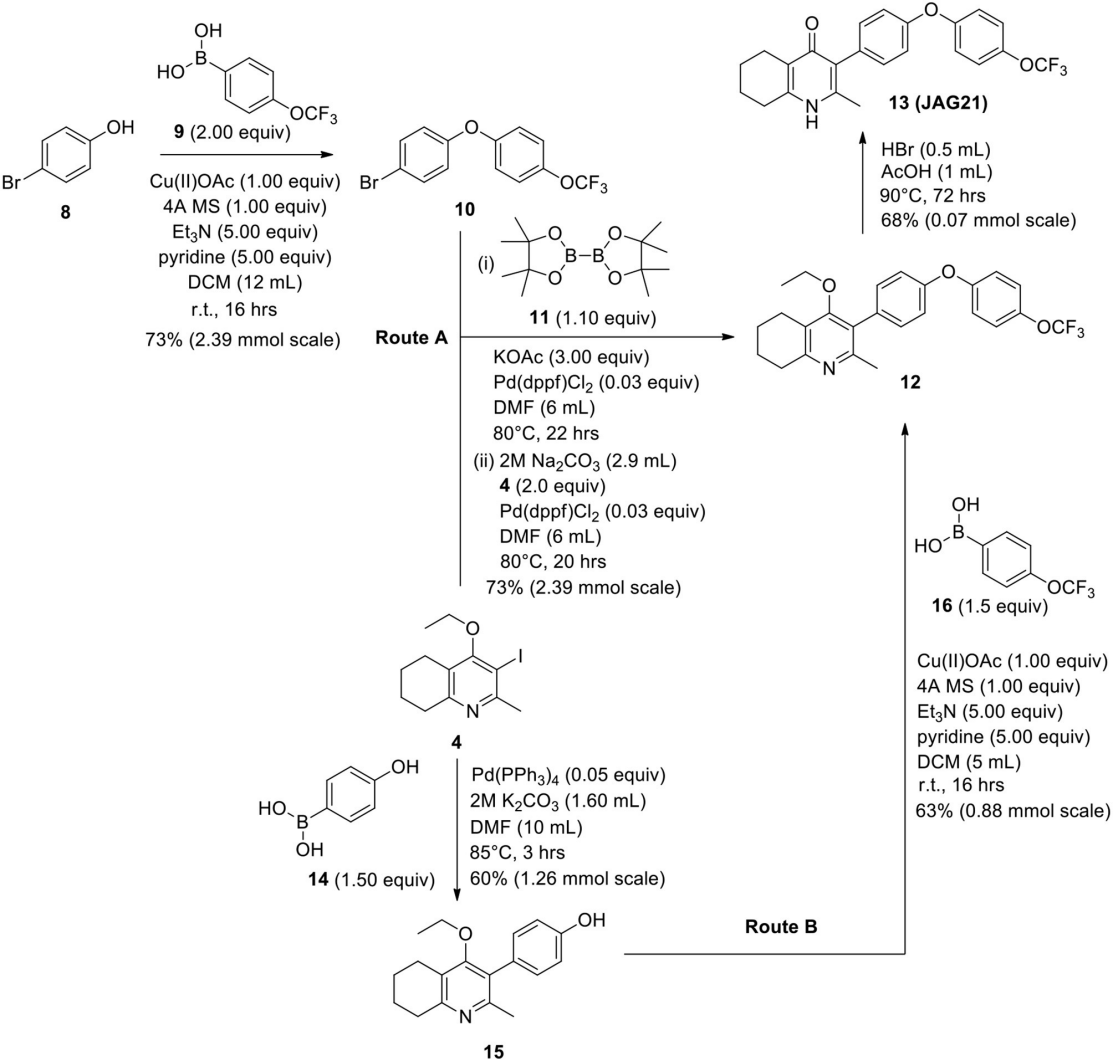
Synthetic route to analogs of 7 (MJM170) via route A or route B. Route A is the original route to analogs but is linear and involves a tricky Suzuki step to intermediate 12 from intermediates 4 and 10. Route B allows quicker access to analogs since intermediate 15 can be made in larger quantities and derivatives can be synthesized via the Chan-Lam reaction to give final intermediate 12 by varying the boronic acid 16.

The THQ compounds were synthesized at the University of Leeds as described below. Ten millimolars stock solutions were made with 100% Dimethyl Sulfoxide (DMSO) [Sigma Aldrich] and working concentrations were made with IMDM-C (1x, [+] glutamine, [+] 25 mM HEPES, [–] Phenol red, 10% FBS)[Gibco, Denmark]). Compounds are shown in Figure 1A. Compound name with “0” or no “0” between letters and number, e.g., JAG21 or JAG021, refer to the same compound. This is throughout the manuscript. Final compounds had >95% purity determined by high performance liquid chromatography (HPLC), high resolution mass spectrometry, and NMR spectrometry. Liquid chromatography-mass spectrometry (LC-MS) and NMR spectrometry were used to determine the integrity and purity of all intermediates. THQ compounds were synthesized as described in Schemes 1, 2, which describe compounds MJM170 and JAG21 as exemplars. Building blocks 1, 8, 9, and 14 were varied to create the complete series (Figure 1A).

#### Synthesis of 2-Methyl-5,6,7,8-Tetrahydroquinolin-4-one (2)


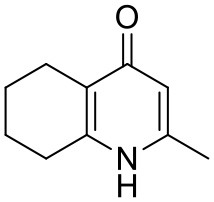


Platinum oxide (0.100 g, 10 mol %) was added to a solution of 4-hydroxy-2-methylquinoline (**1**, 1.00 g, 6.28 mmol, 1.00 eq) in glacial acetic acid (10.0 mL). The heterogeneous mixture was catalytically hydrogenated under a balloon of hydrogen. After 22 h, TLC (10% MeOH–DCM) confirmed complete reaction. The mixture was filtered through celite under vacuum, washing thoroughly with EtOAc (50 mL). The filtrate was concentrated and the resulting residue purified by column chromatography (10% MeOH–DCM) to give the desired product as a pale yellow oil (0.917 g, 5.65 mmol, 89%); ***R***_**f**_ 0.14 (10% MeOH–DCM); **δ_H_** **(300 MHz, CDCl**_**3**_**)** 1.74–1.76 (4H, m, CH_2_), 2.29 (3H, s, Me), 2.49–2.52 (2H, m, CH_2_), 2.67–2.70 (2H, m, CH_2_), 6.16 (1H, s, Ar-H); **δ_C_** **(125 MHz, CDCl**_**3**_**)** 19.0 (Me), 21.8 (CH_2_), 22.1 (CH_2_), 27.1 (CH_2_), 112.5 (CH), 122.4 (Cq), 146.4 (Cq), 147.0 (Cq), 178.3 (Cq); Spectroscopic data consistent with literature values (Bradbury et al., 1993).

#### Synthesis of 2-Methyl-3-iodo-5,6,7,8-Tetrahydroquinolin-4-One (3)


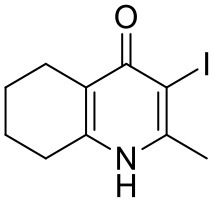


^*n*^Butylamine (6.20 mL, 62.8 mmol, 10.0 eq) was added to a suspension of 2-methyl-5,6,7,8-tetrahydroquinolin-4-one (**2**, 1.02 g, 6.28 mmol, 1.00 eq) in DMF (10.0 mL). To this heterogeneous mixture was added *I*_2_ (1.60 g, 6.28 mmol, 1.00 eq) in a saturated solution of KI (6.00 mL). After 20 h stirring at R.T., a precipitate formed in the orange solution, excess iodine was quenched with 0.1 M sodium thiosulfate solution (10.0 mL). The precipitate was filtered by vacuum filtration, washed with distilled H_2_O and dried (Na_2_SO_4_) to give the desired product as a colorless solid (1.76 g, 6.09 mmol, quantative yield); **δ_H_** **(300 MHz, DMSO-*d***_**6**_**)** 1.61–1.70 (4H, m, CH_2_), 2.29 (2H, t, *J* 6.0, CH_2_), 2.43 (2H, s, CH_2_), CH_3_ under DMSO peak.

#### Synthesis of 2-Methyl-3-Iodo-4-Ethoxy-5,6,7,8-Tetrahydroquinoline (4)


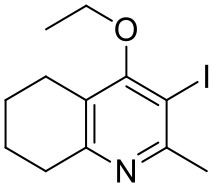


Potassium carbonate (1.53 g, 11.1 mmol, 2.00 eq) was added to a heterogeneous mixture of 2-methyl-3-iodo-5,6,7,8-tetrahydroquinolin-4-one (**3**, 1.60 g, 5.56 mmol, 1.00 eq) in DMF (15.0 mL), and the reaction heated to 50°C for 30 min. The R.B. flask was removed from the heating mantle and ethyl iodide (0.67 mL, 8.33 mmol, 1.50 eq) was added dropwise. The reaction was then heated at 50°C for 18 h. The reaction was cooled to R.T., quenched with water (40 mL). The resulting emulsion formed which was extracted with EtOAc (50 mL). EtOAc layer were washed with water (3 × 30 mL), brine (3 × 30 mL), dried (Na_2_SO_4_) and concentrated to give a pale yellow oil (1.09 g, 3.44 mmol, 61%); ***R***_**f**_ 0.88 (1:1 Pet–EtOAc); **HPLC** (RT = 1.67 min); **LCMS** (Method A), (RT = 1.6 min, *m/z* (ES) Found MH^+^ 318.0); **δ_H_** **(500 MHz, CDCl**_**3**_**)** 1.49 (3H, t, *J* 7.0, ethoxy CH_3_), 1.73–1.78 (2H, m, CH_2_) 1.84–1.88 (2H, m, CH_2_), 2.78–2.69 (5H, m, CH_2_ & CH_3_), 2.84 (2H, t, *J* 6.5, CH_2_), 3.97 (2H, q, *J* 7.0, OCH_2_); **δ_C_** **(125 MHz, CDCl**_**3**_**)** 15.6 (CH_3_), 22.3 (CH_2_), 22.8 (CH_2_), 23.6 (CH_2_), 29.3 (CH_3_), 32.0 (CH_2_), 68.4 (OCH_2_), 90.9 (Cq), 124.5 (Cq), 158.3 (Cq), 158.9 (Cq), 163.9 (Cq).

#### Synthesis of 2-Methyl-3-(4-Phenoxyphenyl)-4-Ethoxy-5,6,7,8-Tetrahydroquinoline (6)


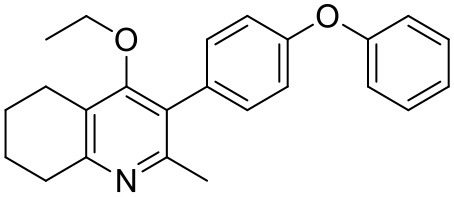


2-Methyl-3-iodo-4-ethoxy-5,6,7,8-tetrahydroquinoline (**4**, 0.266 g, 0.839 mmol, 1.00 eq), Pd(PPh_3_)_4_ (0.048 g, 0.0419 mmol, 5 mol%) and 4-phenoxyphenylboronic acid (**5**, 0.270 g, 1.26 mmol, 1.50 eq) were charged to a R.B. flask under N_2_(g). Degassed DMF (10.0 mL) was added to the flask followed by 2M K_2_CO_3_ (1.60 mL). The flask was heated to 85°C under N_2_(g). After 15 min, TLC (4:1 Pet–EtOAc) confirmed reaction was complete. The reaction was cooled and diluted with EtOAc (15 mL), filtered through celite and partitioned between EtOAc (10 mL) and H_2_O (25 mL). Combined organics were washed with H_2_O (3 × 30 mL), then brine (3 × 30 mL), dried (Na_2_SO_4_) and concentrated to give a red oil which was purified by column chromatography (3:1 Pet–EtOAc), to give the desired product as a pale yellow oil (0.235 g, 0.655 mmol, 78%); ***R***_**f**_ 0.31 (3:1 Pet–EtOAc); **HPLC** (RT = 3.08 min); **δ_H_** **(300 MHz, CDCl**_**3**_**)** 1.04 (3H, t, *J* 7.0, ethoxy CH_3_), 1.76–1.93 (4H, m, 2xCH_2_), 2.32 (3H, s, CH_3_) 2.72 (2H, t, *J* 6.0, CH_2_), 2.91 (2H, t, *J* 6.5, CH_2_), 3.50 (2H, q, *J* 7.0, OCH_2_), 7.05–7.16 (5H, m, Ar-H), 7.20–7.29 (2H, m, Ar-H), 7.31–7.43 (2H, m, Ar-H); **δ_C_** **(125 MHz, CDCl**_**3**_**)** 15.7 (CH_3_), 22.5 (CH_2_), 23.0 (CH_3_), 23.3 (CH_2_), 23.4 (CH_2_), 32.7 (CH_2_), 68.2 (OCH_2_), 118.6 (CH), 118.9 (CH), 123.4 (CH), 126.8 (Cq), 129.8 (CH), 131.5 (CH), 154.9 (Cq), 156.5 (Cq), 157.1 (Cq), 157.3 (Cq); ***m/z* (*ES*)** (Found: MH^+^, 360.1973. C_24_H_26_NO_2_ requires *MH*, 360.1964).

#### Synthesis of 2-Methyl-3-(4-Phenoxyphenyl)-4-Ethoxy-5,6,7,8-Tetrahydroquinoline (7, MJM170)


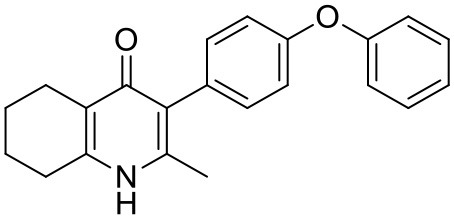


Aqueous hydrobromic acid (>48%) (1.00 mL) was added to a solution of 2-methyl-3-(4-phenoxyphenyl)-4-ethoxy-5,6,7,8-tetrahydroquinoline (**6**, 0.226 g, 0.630 mmol, 1.00 eq) in glacial acetic acid (2 mL). The reaction was stirred at 90°C for 5 days, monitoring by LMCS. The reaction was cooled to R.T. and the pH adjusted to pH 5 with 2M NaOH. The precipitate was collected by vacuum filtration and recrystallized from MeOH:H_2_O to give the desired product as an off-white solid (0.155 g, 0.467 mmol, 74%); **HPLC** (RT = 2.56 min); **δ_H_** **(500 MHz, DMSO-*d***_**6**_**)** 1.66–1.72 (4H, m, 2xCH_2_), 2.08 (3H, s, CH_3_) 2.31 (2H, t, *J* 6.0, CH_2_), 2.56 (2H, t, *J* 6.0, CH_2_), 6.99 (2H, d, *J* 8.5, Ar-H), 7.06 (2H, d, *J* 7.5, Ar-H), 7.14–7.18 (3H, m, Ar-H), 7.40–7.43 (2H, m, Ar-H), 11.0 (1H, s, NH); **δ_C_** **(125 MHz, DMSO-*d***_**6**_**)** 17.7 (CH_3_), 21.5 (CH_2_), 21.8 (CH_2_), 21.9 (CH_2_), 26.2 (CH_2_), 117.8 (CH), 118.6 (CH), 121.2 (Cq), 123.3 (CH), 123.7 (Cq), 130.0 (CH), 131.4 (Cq), 132.3 (CH), 142.3 (Cq), 143.2 (Cq), 155.0 (Cq), 156.8 (Cq), 175.4 (Cq); ***m/z* (*ES*)** (Found: MH^+^, 332.1654. C_22_H_22_NO_2_ requires *MH*, 332.1645).

#### Synthesis of 1-(4-Bromophenyl)-4-(Trifluoromethoxy)Benzene (10)


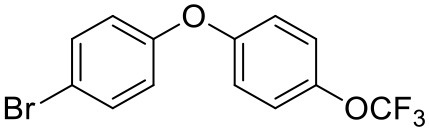


Copper (II) acetate (0.435 g, 2.39 mmol, 1.00 eq) was added to a suspension of 4-bromophenol (**8**, 0.414 g, 2.39 mmol, 1.00 eq), 4-trifluoromethoxybenzeneboronic acid (**9**, 0.983 g, 4.79 mmol, 2.00 eq) and 4 Å molecular sieves (0.566 g) in DCM (12 mL) at R.T. A solution of triethylamine (1.7 mL, 11.9 mmol, 5.00 eq) and pyridine (1 mL, 11.9 mmol, 5.00 eq) was added and the reaction was stirred for 16 h, open to the atmosphere. After 18 h, the reaction was quenched with 0.5 M HCl (20 mL) and the organic layer washed with water (20 mL), brine (20 mL), dried (Na_2_SO_4_), and concentrated to give a red oil which was purified by column chromatography (hexane) to give the desired product as a colorless oil (0.582 g, 1.75 mmol, 73%); ***R***_**f**_ 0.58 (hexane).

#### Synthesis of 2-Methyl-3-(4-Hydroxyphenyl)-4-Ethoxy-5,6,7,8-Tetrahydroquinolin-4-One (15)


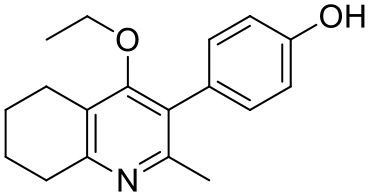


2-Methyl-3-iodo-4-ethoxy-5,6,7,8-tetrahydroquinoline (**4**, 0.400 g, 1.26 mmol, 1.00 eq), Pd(PPh_3_)_4_ (0.073 g, 0.06 mmol, 5 mol%) and 4-hydroxylphenylboronic acid (**14**, 0.260 g, 1.89 mmol, 1.50 eq) were charged to a R.B. flask under N_2_(g). Degassed DMF (10.0 mL) was added to the flask followed by 2M K_2_CO_3_ (3.00 mL). The flask was heated to 85°C under N_2_(g). After 3 h, TLC (EtOAc) confirmed reaction was complete. The reaction was cooled to 50°C, diluted with EtOAc (15 mL) and activated charcoal was added. After stirring for 30 min, the mixture was filtered through celite and partitioned between EtOAc (10 mL) and H_2_O (25 mL). Combined organics were washed with H_2_O (3 × 30 mL), then brine (3 x 30 mL), dried (Na_2_SO_4_) and concentrated to give a brown solid which was triturated with diethyl ether to give the desired product as a pale red crystalline solid (0.220 g, 0.777 mmol, 60%); ***R***_**f**_ 0.22 (EtOAc); **m.p**. 225–226°C (EtOAc); **δ_H_** **(500 MHz, MeOD-*d***_**4**_**)** 7.07 (d, *J* = 8.6 Hz, 2H, H-3 & 5), 6.86 (d, *J* = 8.6 Hz, 2H, H-2 & 6), 3.51 (q, *J* = 7.0 Hz, 2H, CH_3_C*H*_2_O), 2.83 (t, *J* = 6.3 Hz, 2H, H-8'), 2.72 (t, *J* = 6.1 Hz, 2H, H-5'), 2.23 (s, 3H, Me), 1.95–1.72 (m, 4H, H-6' & 7'), 1.00 (t, *J* = 7.0 Hz, 3H, C*H*_3_CH_2_O); **δ_C_** **(125 MHz, MeOD-*d***_**4**_**)** 164.0 (Cq), 158.1 (C-1), 157.4 (C_q_), 156.1 (C_q_), 132.2 (C-3 & 5), 129.1 (Cq), 127.9 (Cq), 124.9 (Cq), 116.2 (CH), 69.1 (OCH_2_), 32.7 (CH_2_), 23.9 (CH_2_), 23.4 (CH_3_), 22.9 (CH_2_), 22.3 (CH_2_), 15.7 (CH_3_); ***m/z* (*ES*)** (Found MH^+^, 284.1664, C_18_H_21_NO_2_ requires *MH*, 284.1651).

#### Synthesis of 2-Methyl-3-(4-Hydroxyphenyl)-4-Ethoxy-5,6,7,8-Tetrahydroquinolin-4-One (12)


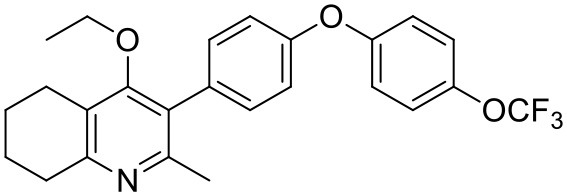


1-(4-bromophenyl)-4-(trifluoromethoxy)benzene (**10**, 0.100 g, 0.30 mmol, 1.00 eq), bis(pinacolato) diboron (1.10 eq), potassium acetate (3.00 eq) and Pd(dppf)Cl_2_ (0.03 eq) were added to a oven-dried flask under inert (N_2_) atmosphere. Anhydrous DMF (6 mL) was added and the reaction heated to 80°C under N_2_ (g). After 22 h, the reaction was cooled to R.T., fresh Pd(dppf)Cl_2_ (0.03 eq) added, followed by 2-methyl-3-iodo-4-ethoxy-5,6,7,8-tetrahydroquinoline (**4**, 0.400 g, 1.26 mmol, 2.00 eq) and 2M Na_2_CO_3_ (2.9 mL). The reaction was heated to 80°C for 20 h, cooled, diluted with EtOAc (20 mL), filtered through celite and partitioned between EtOAc (20 mL) and H_2_O (20 mL). Combined organics were washed with brine (3 × 20 mL), dried (Na_2_SO_4_) and concentrated to give a brown solid which was purified by column chromatography (3:1 Pet–EtOAc) to give the desired product as a colorless oil (30 mg, 0.07 mmol, 23%); **HPLC** (RT = 2.41 min); **δ_H_** **(500 MHz, acetone)** 7.28 (d, *J* = 8.7 Hz, 2H, H-2′ & 6′), 7.26 (d, *J* = 9.1 Hz, 2H, H-2″ & 6″), 7.09 (d, *J* = 9.1 Hz, 2H, H-3″ & 5″), 7.07 (d, *J* = 8.7, 2H, H-3′ & 5′), 3.52 (q, *J* = 7.0 Hz, 2H, CH_3_*CH*_2_O), 2.85 (t, *J* = 6.5 Hz, 2H, H-8), 2.78 (t, *J* = 6.2 Hz, 2H, H-5), 2.26 (s, 3H, Me), 1.89–1.81(m, 2H, H-7), 1.81–1.72 (m, 2H, H-6), 0.93 (t, *J* = 7.0 Hz, 3H, C *H*_3_CH_2_O); **δ_C_** **(125 MHz, acetone)** δ 161.9 (Cq), 157.1 (Cq), 156.5 (Cq), 156.0 (Cq), 154.5 (Cq), 145.3 (Cq), 132.5 (Cq), 132.0 (CH), 126.7 (Cq), 123.0 (CH), 119.8 (CH), 119.0 (CH), 68.0 (OCH_2_), 32.5 (CH_2_), 23.0 (CH_2_), 22.9 (CH_3_), 22.7 (CH_2_), 22.5 (CH_2_), 15.05 (CH_3_); ***m/z* (*ES*)** (Found: MH^+^, 444.1792. C_25_H_24_F_3_NO_3_ requires *MH*, 444.1781).

#### Synthesis of 2-Methyl-3-(4-(4-(Trifluoromethoxy)Phenoxy)Phenyl)-5,6,7,8-Tetrahydroquinolin-4-One (13, JAG21)


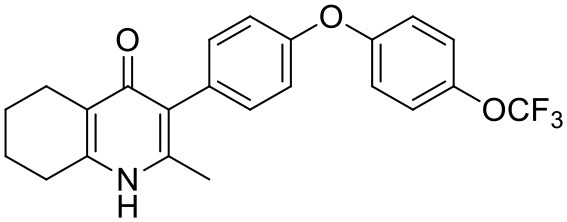


Aqueous hydrobromic acid (>48%) (1.00 mL) was added to a solution of 2-methyl-3-(4-phenoxyphenyl)-4-ethoxy-5,6,7,8- tetrahydroquinoline (**12**, 30.0 mg, 0.07 mmol, 1.00 eq) in glacial acetic acid (2 mL). The reaction was stirred at 90°C for 3 days, monitoring by LMCS. The reaction was cooled to R.T. and the pH adjusted to pH 5 with 2M NaOH. The precipitate was collected by vacuum filtration and recrystallized from MeOH:H_2_O to give the desired product as a colorless solid (25.0 mg, 0.06 mmol, 68%); **m.p**. >250°C; **HPLC** (RT = 2.78 min); **δ_H_** **(500 MHz, DMSO-*d***_**6**_**)** 11.07 (s, 1H, NH), 7.40 (d, J = 8.5 Hz, 2H, H-2′ & 6′), 7.19 (d, *J* = 8.6 Hz, 2H, H-3″ & 5″), 7.13 (d, *J* = 9.0 Hz, 2H, H-3′ & 5′), 7.02 (d, J = 8.5 Hz, 2H, H-2″ & 6″), 2.54 (t, J = 6.0 Hz, 2H, H-8), 2.28 (t, J = 5.9 Hz, 2H, H-5), 2.07 (s, 3H, Me), 1.71 (m, 2H, H-7), 1.65 (m, 2H, H-6); **δ_C_** **(125 MHz, DMSO-*d***_**6**_**)** 175.7 (Cq), 155.9 (Cq), 154.5 (Cq), 143.5 (Cq), 143.2 (Cq), 142.2 (Cq), 132.5 (CH), 132.2 (Cq), 123.6 (Cq), 123.0 (CH), 121.3 (Cq), 119.6 (CH), 118.2 (CH), 26.2 (CH_2_), 21.9 (CH_2_) 21.8 (CH_2_), 21.5 (CH_2_), 17.7 (CH_3_); ***m/z* (*ES*)** (Found: MH^+^, 416.1492. C_23_H_20_F_3_NO_3_ requires *MH*, 416.1473).

### Toxoplasma gondii

#### Parasite Strains (Isolates)

RH-YFP tachyzoites (Gubbels et al., 2003; Fomovska et al., 2012a; McPhillie et al., 2016), EGS strain (Vidigal et al., 2002; Paredes-Santos et al., 2013, 2018; McPhillie et al., 2016), Pru-luciferase, Me49, and RPS13Δ on the RH strain background (Hutson et al., 2010) were prepared and passaged in human foreskin fibroblasts [HFF] as described.

#### T. gondii in vitro

##### *In vitro* Challenge Assay for *T. gondii*

*RH strain YFP Tachyzoites*. Protocol was adapted from Fomovska et al. (2012a,b) for HFF. HFF were cultured on a flat, clear-bottomed, black 96-well plate to 90–100% confluence. IMDM (1x, [+] glutamine, [+] 25 mM HEPES, [+] Phenol red, 10% FBS [Gibco, Denmark]) was removed and replaced with IMDM-C(1x, [+] glutamine, [+] 25 mM HEPES, [–] Phenol red, 10% FBS)[Gibco, Denmark]). RH-YFP, lysed from host cells by passing twice through a 27-gauge needle, were counted, then diluted to 32,000/mL in IMDM-C. HFF were infected with 3200 RH-YFP, then returned to 37°C, CO_2_ (5%) incubator for 1–2 h for infection. Various concentrations of the compounds in 20 μL IMDM-C were added to each well. There were triplicates for each condition. Controls were pyrimethamine/sulfadiazine (standard treatment), 0.1% DMSO only, HFF only, and untreated cultures of HFF infected with 2-fold dilutions of YFP expressing parasites (called “YFP gradient” to establish amount of color from known numbers of YFP expressing parasites). Cells were incubated at 37°C for 72 h. Plates were read using a fluorimeter (Synergy H4 Hybrid Reader, BioTek) to ascertain amount of relative fluorescence units (RFU) YFP, to measure parasite burden after treatment. Data were collected using Gen5 software with IC_50_ calculated by graphical analysis in Excel.

An initial screening assay of 10 μM, 1 μM, 100 nM, and 10 nM of the compounds was performed. Compounds were not considered effective or pursued for further analysis if there was no inhibition of tachyzoites at 1 μM. If compounds were effective at 1 μM, another experiment was performed to assess effect at 1 μM, 500, 250, 125, 62.5, and 31.25 nM.

#### Cytotoxicity Assays in Parallel With RH Strain *T. gondii in vitro* Studies

Toxicity assays used WST-1 cell proliferation reagent (Roche) as in Fomovska et al. (2012a). HFF were grown on a flat, clear-bottomed, black 96-well plate. Confluent HFF were treated with inhibitory compounds at concentrations of 10 and 50 μM. Compounds were diluted in IMDM-C, and 20 μL were added to each designated well, with triplicates for each condition. A gradient with 2-folddecreasing concentrations of DMSO from 10 to 0% in colorless, translucent IMDM-C was used as a control. The plate was incubated for 72 h at 37°C. Ten microlitre WST-1 reagent (Roche) were added to each well. Cells were incubated for 30–60 min. Absorbance was read using a fluorimeter at 420 nm. A higher degree of color change (and absorbance) indicated mitochondrial activity and cell viability.

#### *In vitro* Challenge Assay for EGS Strain Bradyzoites

HFF cells were grown in IMDM on removable, sterile glass cover slips in the bottom of a clear, flat-bottomed 24-well plate. Cultures were infected with 3 × 10^4^ EGS strain parasites per well, in 0.5 mL media. The plate was returned to incubator at 37°C overnight. The following day, the media was removed. Colorless IMDM and compounds were added to make various concentrations of the drug. Total volume was 0.5 mL. Two wells had media only, as a control. Plates were returned to the 37°C incubator for 72 h, checked once each 24 h. If tachyzoites were visible in the control before 72 h, cells were fixed and stained.

Cells were fixed using 4% paraformaldehyde and stained with Fluorescein-labeled Dolichos Biflorus Agglutinin, DAPI, and antibody to BAG1. Disks were removed, mounted on glass slides, and visualized using microscopy (Nikon Tl7). Slides were scanned using a CRi Panoramic Scan Whole Slide Scanner and viewed using Panoramic Viewer Software. Effects of compounds were quantitated by counting cysts in controls and treated cultures. Dolichos staining delimited structures and single organisms that remained were counted in a representative field of view. This was then multiplied by a factor determined by the total area of the cover slip in order to estimate the number of cysts and organisms in each condition. When the following forms were observed: “true cysts” with a dolichos-staining wall, “pseudocysts” or tight clusters of parasites, and small organisms, if there were fewer than four parasites visible in a cluster, organisms were counted individually (as “small organisms”). The entire scanned coverslip with all fields was also reviewed by 3 observers to confirm consistency.

#### Synergy Studies With RH Strain YFP Tachyzoites

Atovaquone and pyrimethamine were used to test whether they are synergistic with JAG21. Serial dilutions of the combination of JAG21 and either atovaquone or pyrimethamine were used in an *in vitro* challenge assay as described above. The EC50 of each compound and the combination of two compounds were determined. The effect of the combination of drugs was calculated with the following formula:C= [A]c/[A]a+[B]c/[B]a. If C is lower than 1, the two compounds tested have synergistic effect; if C is >1, the two compounds tested have antagonist effect and if C is 1 they are additive.

#### *T. gondii* and HFF Mitochondrial Membrane Potential Measurements

The mitochondrial membrane potential was measured by the safranine method according to Vercesi et al. (1998). Freshly egressed *T. gondii* tachyzoites were filtered and washed twice with intracellular buffer (125 mM sucrose, 65 mM KCl, 10 mM HEPES-KOH buffer, pH 7.2, 1 mM MgCl_2_, and 2.5 mM potassium phosphate). After washing, the parasites were resuspended in the same buffer at 10^9^/mL. An aliquot of 50 μL of this suspension was added to a cuvette containing Safranin O, 2.5 μM and Succinate 1 mM in final volume of 2 mL of the intracellular buffer. The fluorescence was measured with a Hitachi 7000 spectrofluorometer with settings Ex. 495/Em. 586. Once the baseline fluorescence was stable, 30 μM digitonin was added to permeabilize the parasites. Eighty five seconds after permeabilization, the THQ derivatives, dissolved in DMSO, were added. Five micromolars of FCCP (Carbonyl cyanide-4-(trifluoromethoxy) phenylhydrazone) was used as an uncoupler reference for calculations and its effect was considered 100%. We used similar conditions for measuring the mitochondrial membrane potential of mammalian cells with the following changes: the mammalian cells were resuspended at 10^8^/mL. We also used 50 μL of this suspension for each experiment in a total volume of 2 mL. The substrate used for mammalian cells was 5 mM glutamate and 5 mM malate. A higher concentration of digitonin (50 μM) was used to permeabilize the mammalian cells. The compounds were added at ~400 s after permeabilization. Each experiment was repeated at least three times in duplicates. Statistical analysis, unpaired student *t*-test, was performed using GraphPad Prism 8.0 (GraphPad Software, Inc., San Diego, CA).

#### Structure Activity Relationship (SAR) and Comparison of Effect on Toxoplasma gondii and HFF Enzyme Activity

The effects of changing R1 as 7-Et, 7-Me, 6-CF_3_, or 6-Me on activity against RH strain tachyzoites, kinetic solubility, and metabolic stability were compared. Kinetic solubility and metabolic stability in human or murine liver microsomes were measured. The hERG (human Ether-à-go-go-Related) liability was also determined. The hERG gene (KCNH2) encodes a protein K_v_11.1, the alpha subunit of a potassium ion channel. This channel conducts the rapid component of the delayed rectifier potassium current, IKr, which is critical for repolarization of cardiac action potentials. A reduction in hERG currents from adverse drug effects can lead to long QT interval syndromes. These syndromes are characterized by action potential prolongation, lengthening of the QT interval on surface EKG, and an increased risk for “torsade de pointes” arrhythmias and sudden death. The MDCK-MDR1 Permeability Assay was also performed. MDCK-MDR1 refers to the ability of a compound to permeate across membranes of MDCK-MDR1 (Madin Darby canine kidney [MDCK] cells with the *MDR1* gene [ABCB1], the gene encoding for the efflux protein, P-glycoprotein (*P-gp*)) *in vitro*. Assessing transport in both directions (apical to basolateral and basolateral to apical) across the cell monolayers enables an efflux ratio to be determined. This provides an indication as to whether a compound undergoes active efflux (mediated by P-*gp*). This provides a prediction of blood brain barrier (BBB) penetration potential/permeability and efflux ratio. Effect in CACO-2 (Colon Adenocarcinoma cells) as a permeability assay and on cytochrome P450 (CYP 450) were also determined. CYP enzymes catalyze oxidative biotransformation (phase 1 metabolism) of most drugs. CYP enzymes, bind to membranes in a cell (cyto) and contain a heme pigment (chrome and P) that absorbs light at a wavelength of 450 nm when exposed to carbon monoxide. Metabolism of a drug by CYP enzymes is a major source of variability in drug effect. These were measured by Chem Partners. The relative effect on HFF and parasite enzymes also were compared.

#### RPS13Δ Tachyzoites in Human Primary Brain Neuronal Stem Cells *in vitro* for Transcriptomics and Transcriptomics Analyses

Culture of Human Primary Brain Neuronal Stem Cells (NSC) was as described (McPhillie et al., 2016; Ngô et al., 2017); *T. gondii* RPS13Δ on RH strain background (Hutson et al., 2010) was used to infect the NSC as described (McPhillie et al., 2016; Ngô et al., 2017). RNA was isolated and prepared and used for transcriptomic experiments as described (McPhillie et al., 2016; Ngô et al., 2017). Briefly, NSC, initially isolated from a temporal lobe biopsy (Walton et al., 2006) were infected with either wild-type or RPS13Δ RH tachyzoites using biological duplicates at a multiplicity of infection of 2:1 and incubated as previously described Ngô et al. (2017). Eighteen hours post-infection, extracellular parasites were washed out with cold PBS before total RNA extraction. Further isolation of the mRNA fraction was carried out with miRNeasy Mini Kit columns (Qiagen) following manufacturer instructions and Illumina barcoded mRNA sequencing libraries were constructed with TruSeq RNA Sample Preparation Kits v2 (Illumina). Libraries were sequenced as 100 bp single reads with Illumina HiSeq 2000 apparatus at a sequencing depth of ~3 Gbp per sample. Sequencing reads were mapped to the human (release GRCh38) and *T. gondii* ME49 strain (ToxoDB release 13.0) reference genome assemblies with hisat2 (Kim et al., 2015) and raw read counts were per gene were estimated with HTSeq (Anders et al., 2015). Identification of parasite genes that were differentially expressed between wild-type and RPS13Δ parasites was performed with the R package DESeq2 (Love et al., 2014) using a generalized linear model likelihood ratio test. Identification of orthologous genes between *T. gondii* and *P. cynomolgi* was carried out by best-reciprocal matches between *T. gondii* and *P. cynomolgi* proteomes using Blastp and a e-value cutoff of 1 × 10^−3^. The list of Genes that are differentially expressed between *P. cynomolgi* hypnozoites and the liver-schizont stage was extracted from a previously published study by Cubi et al. (2017). Gene set enrichment analysis was carried out with the GSEA tool (Subramanian et al., 2005) using *T. gondii* Gene Ontology and cell cycle gene sets developed by Croken et al. (2014) and visualized with the Enrichment Map application in Cytoscape (Su et al., 2014).

### Toxoplasma gondii in vivo

#### Type II Parasites *in vivo*

##### IVIS

Balb/C mice were infected intraperitoneally (IP) with 20 × 10^3^ *T. gondii* (Prugneaud strain expressing luciferase) tachyzoites. Treatment began 2 h later with JAG21 (5 mg/kg) which was dissolved in DMSO, administered IP in a total volume of 0.05 mL. Mice were imaged every second day starting on day 4 post infection using an IVIS Spectrum (Caliper Life Sciences) for minute exposures, with medium binning, 20 min post injection with 150 mg/kg of D-luciferin potassium salt solution.

##### Brain cysts

Brain cysts were searched for in paraffin imbedded tissue of the surviving Prugneaud strain infected treated Balb/C mice in the IVIS study, 30 days after infection which was 16 days after treatment had been discontinued. All treated mice had survived. There were no surviving untreated mice in those experiments.

In separate experiments, Balb/C mice were infected IP with 20 × 10^3^ *T. gondii* Me49 strain tachyzoites. In these separate studies of mice with established chronic infection, after 30 days, IP treatment with JAG21 was begun each day for 14 days. JAG21 was dissolved in DMSO and administered IP in a total volume of 0.05 mL. In experiments when tafenoquine was administered alone or with JAG21 in some groups 3 mg/kg tafenoquine was administered once on day−1 from when JAG21 treatment was initiated. Cysts in brain were quantitated on day 30, 16 days after discontinuing JAG21. Immunoperoxidase staining was performed. Parasite burden was quantitated in two ways. The first was using a positive pixel count algorithm of Aperio ImageScope software. Positive pixels were normalized to tissue area (mm^2^). Briefly, automated quantitation was done by counting positive pixels per square area. The entire brain in one section was scanned for each mouse. The Cyst burden was quantitated as units of positive pixels per mm^2^. The average ± S.E.M. numbers of mm^2^ per slide quantitated was 30.2±1.6 square mm per mouse for this quantification. Each highpower field of view shown in Figure 5C is ~0.02 mm^2^ per field of view. Cysts on each slide for each condition in two biological replicate experiments were also quantitated by 2 separate observers independently and results compared with automated counting, separately.

### RPS13 Δ *in vivo*

This G1 arrested parasite persists in tissue culture for prolonged times in the absence of tetracycline (Hutson et al., 2010), but in immune competent mice it cannot be rescued with teteracycline, or LNAME (L-N^G^-Nitro arginine methyl ester, an analog of arginine) used as an antagonist of nitric oxide synthase (NOS) that inhibits NO production, or both together (Hutson et al., 2010).

In pilot studies, herein, interferon γ receptor knockout mice that were not treated were observed following infection. At 7 and at 14 days following infection, spleen, and liver were removed and immune peroxidase stained. At 14 days a group of mice were treated with anhydrotetracycline and when a subset of these mice died, their spleen and liver were removed and immune peroxidase stained.

As in the pilot studies, this RPS13 Δ parasite also was used to infect interferon γ receptor knockout mice in a treatment study. The design of this experiment with these immune compromised mice is shown in Figure 6. In this separate study, groups of mice were infected with RPS13 Δ. They were treated with tafenoquine on day−1, or JAG21 for 14 days 2 h after infection, or the two together with tafenoquine on day−1 and JAG21 for the first 14 days, or with diluent only for 14 days, as described above. For the initial 14 days, no tetracycline was administered. After that time tetracycline was administered. Mice were observed each day. At the time they appeared to have substantial illness or at the termination of the experiment they were euthanized, tissues fixed in formalin and stained with hematoxylin and eosin or immunoperoxidase stained and parasite burden was assessed.

#### RH Challenge in a Study of Oral Administration of a Novel Nano Formulation of JAG21

##### Nanoformulation of JAG21 for oral administration in *T. gondii* studies

JAG21 was prepared using hydroxyethyl cellulose (HEC) and Tween 80. Briefly, this dispersant solution containing 5 mg/mL HEC and 2 mg/mL Tween 80 in water was prepared. Solid JAG21 was added to 20 mg/mL, and the dispersion was sonicated for 60 s using a Sonics vc50 probe-tip sonicator set to 20 kHz to homogenize. Sonication was performed at room temperature. Aliquots of the homogeneous dispersion were frozen and lyophilized using a VirTis AdVantage freeze drier. These aliquots were stored at room temperature for 5–6 months. Prior to dosing, aliquots were reconstituted using water. Controls containing no JAG21 were also prepared. Following reconstitution with water, the dispersion was imaged using a Nikon ECLIPSE E200 optical microscope set to 40x magnification. The average particle size of the JAG21 dispersion in HEC/Tween 80 was determined using an in-house image analysis program. This novel method to stably formulate JAG21 was discovered after all other studies were completed and this was the last experiment in this manuscript performed as a consequence.

##### RH YFP challenge

For studies of the nano formulated JAG 21, this was administered for 1 or 3 days by gavage in the doses shown in the results section. These C57BL6 background mice received 2000 RH tachyzoites IP. on day the first day of the experiment and peritoneal fluid was collected 5 days later to quantitate fluorescence and numbers of parasites.

### Malaria Assays

#### Enzyme Assays

##### Methods for enzyme assays: Materials

P. falciparum 3D7 strain were obtained from the Liverpool School of Tropical Medicine. Protease cocktail inhibitor was obtained from Roche. Bradford protein assay dye reagent was obtained from Bio-Rad. All other reagents were obtained from Sigma-Aldrich. Decylubiquinol was produced as per Fisher et al. (2009). In brief, 25 mg of decylubiquinone were dissolved in 400 μL of nitrogen-saturated hexane. An equal volume of aqueous 1 M sodium dithionite was added, and the mixture vortexed until colorless. The organic phase containing the decylubiquinol was collected, the solvent was evaporated under N_2_ and the decylubiquinol finally dissolved in 100 μL of 96% ethanol (acidified with 10 mM HCl). Concentrations of decylubiquinol was determined spectrophotometrically on a Cary 300 Bio UV/visible spectrophotometer (Varian, UK) from absolute spectra, using ε_288−−320_ = 8.1 mM^−1^.cm^−1^. Decylubiquinol was stored at −80°C and used within 2 weeks.

##### P. falciparum culture and extract preparation

P. falciparum strain 3D7 blood-stage cultures were maintained by the method of Trager and Jensen (1976). Cultures contained a 2% suspension of O+ human erythrocytes in RPMI 1640 medium containing L-glutamine and sodium carbonate, and supplemented with 10% pooled human AB+ serum, 25 mM HEPES (pH 7.4) and 20 μM gentamicin sulfate. Cultures were grown under a gaseous headspace of 4% O_2_ and 3% CO_2_ in N_2_ at 37°C. Cultures were grown to a parasitemia of 5% before use.

The protocol for the preparation of parasite extract was adapted from Fisher et al. (2009). Free parasites were prepared from infected erythrocytes pooled from five T75 flasks, by adding 5 volumes of 0.15% (w/v) saponin in phosphate-buffered saline (137 mM NaCl, 2.7 mM KCl, 1.76 mM K_2_HPO_4_, 8.0 mM Na_2_HPO_4_, 5.5 mM D-glucose, pH 7.4) for 5 min, followed by three washes by centrifugation in RPMI containing HEPES (25 mM), and a final resuspension in potassium phosphate buffer (50 mM K_2_HPO_4_, 50 mM KH_2_PO_4_, 2 mM EDTA, pH7.4) containing a protease inhibitor cocktail (Complete Mini; Roche). Parasite extract was then prepared by disruption with a sonicating probe for 5 s, followed by a 1 min rest period on ice to prevent the sample overheating. This process was performed three times. The parasite extract was used immediately. The protein concentration of the parasite extract was determined by Bradford protein assay (Bio-Rad).

##### Pfbc_1_ native assay

P. falciparum bc_1_ complex cytochrome c reductase (*Pfbc*_1_) activity was measured by monitoring cytochrome c reduction at 550 vs. 542 nm using a Cary 300 Bio UV-Visible Spectrophotometer (Varian, UK), using a protocol adapted from Fisher et al. (2009). The assay was performed in potassium phosphate buffer in a quartz cuvette and in a final volume of 700 μL. Potassium cyanide (10 μM), oxidized cytochrome c (30 μM), parasite extract (100 μg protein), and compound/DMSO were added sequentially to the cuvette, with mixing between each addition. Test compounds were added to a final concentration of 1 μM. DMSO (0.1% v/v) and atovaquone (1 μM), a known malarial cytochrome bc_1_ complex inhibitor, were used as negative and positive controls, respectively. The reaction was initiated by the addition of 50 μM decylubiquinol and allowed to proceed for 3 min.

#### Malaria Parasite *in vitro* Studies

Malaria potency testing *in vitro* was performed using 4 different *P. falciparum* strains, D6, TM91-C235, W2, and C2B. The D6 strain is a drug sensitive strain from Sierra Leone, the TM91-C235 strain is a multi-drug resistant strain from Thailand, the W2 strain is a chloroquine resistant strain from Thailand, and the C2B strain is a multi-drug resistant strain with resistance against atovaquone. These assays were performed as described below.

##### Compound Activity against Plasmodium falciparum

Compound activity against *P. falciparum*, was tested using the Malaria SYBR Green I–Based Fluorescence (MSF) Assay. The complete method for performing this microtiter assay is described in previous work published by Plouffe et al. (2008) and Johnson et al. (2007). In brief, this assay uses the binding of the fluorescent dye SYBR Green I to malaria DNA to measure parasite growth in the presence of 2-fold diluted experimental or control. The relative fluorescence of the intercalated SYBR Green I proportional to parasite growth, and inhibitory compounds will result in lower observed fluorescence compared to untreated parasites.

##### Cytotoxicity assays in parallel with *P.falcipaum* assays *in vitro*

Toxicity studies also were performed with HepG2 cells (human liver cancer immortal cell line derived from the liver tissue of a 15-year-old African American, ATCC ^R^ HB-8065^TM^) in parallel with the studies of *P. falciparum*, with inhibitors *in vitro*, as described in McPhillie et al. (2016).

#### *P. berghei* Causal Prophylaxis *in vivo* Model

*P. berghei* sporozoites were obtained from laboratory-reared female *Anopheles stephensi* mosquitoes which were maintained at 18 degrees C for 17–22 days after feeding on a luciferase expressing *P. berghei* infected Swiss CD1ICR. Using a dissecting microscope, the salivary glands were extracted from malaria-infected mosquitoes and sporozoites were obtained. Briefly, mosquitoes were separated into head/thorax and abdomen. Thoraxes and heads were triturated with a mortar and pestle and suspended in medium RPMI 1640 containing 1% C57BL/6 mouse serum (Rockland Co, Gilbertsville, PA, USA). 50–80 heads with salivary glands were placed into a 0.5 mL Osaki tube on top of glass wool with enough dissection media to cover the heads. Until all mosquitoes had been dissected, the Osaki tube was kept on ice. Sporozoites that were isolated from the same batch of mosquitoes were inoculated into C57BL/6, 2D knock-out, and 2D knock-out/2D6 knock-in C57BL/6 mice on the same day to control for biological variability in sporozoite preparations. On day 0, each mouse was inoculated intravenously in the tail vein with ~10,000 sporozoites suspended in 0.1 mL volume. They were stained with a vital dye containing fluorescein diacetate (50 mg/mL in acetone) and ethidium bromide (20 μg/mL in phosphate buffered saline; Sigma Chemical Co, St. Louis, MO, USA) and counted in a hemocytometer to ensure that inoculated sporozoites were viable following the isolation procedure. Viability of the sporozoites ranged from 90 to 100%.

##### Animals

The mice used in these experiments were albino C57BL/6 female mice which were housed in accordance with the current Guide for the Care and Use of Laboratory Animals (1996) under an IACUC approved protocol. All animals were quarantined for 7 days upon arrival, and the animals were fed standard rodent maintenance food throughout the study.

##### Test compounds, homogenization of JAG21 creating a nanoformulation, and administration

Animals were dosed with experimental compounds based on body weight. The suspension solution of orally administered drugs were conducted in 0.5% (w/v) hydroxyethyl cellulose and 0.2% Tween 80 in distilled water. To insure the size of the compounds in the dosing solution were under 50 μM (measured they were 4–6 μM), the suspension was homogenized using a homogenizer (PRO Scientific Inc, Monroe, CT, USA) with a 10 mm open-slotted generator running at 20,000–22,000 rpm for 5 min in an ice bath. The compounds were made fresh each day and used immediately (always in <1/2 h). Stability beyond that time was not determined. It was not anticipated that they would be stable beyond that time.

Compounds were administered on 3 consecutive days (−1, 0, +1) relative to sporozoite infection or a single dose on day 0. Drug suspensions were administered to mice by oral gavage using an 18 gauge intragastric feeder. For the 3 day dosing regimen, compounds were administered at 0.625 mg/kg and for the single dose regimen administered on day 0, compounds were administered at 2.5 mg/kg.

##### *In vivo* imaging

All of the *in vivo* bioluminescent imaging methods utilized have been described previously. Briefly, JAG21 was administered orally on days −1, 0, and 1 with respect to sporozoite inoculation. All inoculated mice were imaged using the Xenogen IVIS-200 Spectrum (Caliper Life Sciences, Hopkinton, MA, USA) IVIS instrument at 24, 48, and 72 h post-sporozoite infection. The bioluminescent imaging experiments were conducted by IP injection of the luciferase substrate, D-Luciferin potassium salt (Xenogen, California and Goldbio, St Louis, MO, USA), into mice at a concentration of 200 mg/kg 15 min before bioluminescent images were obtained. Three minutes after luciferin administration the mice were anesthetized using isoflurane, and the mice were positioned ventral side up on a 37°C platform with continual anesthesia provided through nose cone delivery of isoflurane. All bioluminescent images were obtained using 5 min exposures with f-stop = 1 and large binning setting. Photon emission from specific regions was quantified using Living Image® 3.0 software (Perkin Elmer).

Additionally, blood stage parasitemia was assessed 3 days after imaging was completed by treating small quantities of blood obtained from tail bleeds with the fluorescent dye Yoyo-1 measured by using a flow cytometry system (FC500 MPL, Beckman Coulter, Miami, FL, USA) (Pybus et al., 2013; Marcsisin et al., 2014).

### Methods for Co-crystallization and Binding Studies

#### Bovine Cytochrome bc_1_ Activity Assays

Bovine cytochrome *bc*_1_ inhibition assay was carried out in 50 mM KPi pH 7.5, 2 mM EDTA, 10 mM KCN, 30 μM equine heart cytochrome *c* (Sigma Aldrich), and 2.5 nM bovine cytochrome *bc*_1_ at room temperature. 20 mM inhibitors dissolved in DMSO were added to the assay at a desired concentration without prior incubation. The working concentration of DMSO in the assay did not exceed 0.3% v/v. The reaction was initiated by the addition of 50 μM decylubiquinol (Abcam). The reduced cytochrome *c* was monitored by the different absorption between 550 and 542 nm using extinction coefficient of 18.1 mM^−1^ cm^−1^ in a SPECTRAmax Plus 384 UV-visible Spectrometer. The initial kinetic rate is determined as a zero-order reaction and used as the specific activity of cytochrome *bc*_1_.

#### Bovine Cytochrome bc1 Purification Protocol

##### Preparation of crude mitochondria

Whole fresh bovine heart was collected after slaughter and transported in ice. All work was carried out at 4°C. Lean heart muscle was cut into small cubes and homogenized in the buffer composed from 250 mM sucrose; 20 mM K_2_HPO_4_; 2 mM succinic acid; 0.5 mM EDTA. Buffer was added at a ratio of 2.5 L per 1 kg of muscle tissue. Ph of resulting homogenate was adjusted to 7.8 using 2 M Tris and PMSF protease inhibitor was added to 0.1 mM concentration. The homogenate was then centrifuged in a Sorvall GS-3 rotor at 5,000 g for 20 min. The resulting supernatant was then transferred to a Sorvall GSA rotor and centrifuged at 20,000 g for 20 min. Obtained mitochondrial pellet was washed in 50 mM KPi (pH 7.5); 0.1 mM PMSF buffer before second centrifugation under the same condition. The pellet was collected and sored at −80°C for further use.

#### Solubilization of Membrane Proteins

The frozen mitochondria were thawed and re-suspended in 50 mM KPi (pH 7.5); 250 mM NaCl; 0.5 mM EDTA; 0.1 mM PMSF buffer; a small sample was taken for quantification of total mitochondrial proteins by BCA assay. The remaining sample was centrifuged at 180,000 g in Beckman Ti70 rotor for 60 min. The pellet was re-suspended in the same wash buffer with the addition 1 mg DDM per 1 mg of protein and then centrifuged under the same conditions for 60 min. The pellet was discarded and the supernatant was collected for ion exchange chromatography.

#### Purification of Cytochrome bc1

During purification the presence of protein was detected using 280 nm absorbance and the presence of heme was detected using 415 nm Soret band peak and 562 nm absorbance. The solubilized protein solution was applied on DEAE-Sepharose CL-6B column (ca. 50 mL, GE Healthcare) pre-equilibrated with buffer A [50 mM KPi (pH 7.5); 250 mM NaCl; 0.01% w/v DDM; 0.5 mM EDTA] and washed with 3 CV of buffer A. The protein was eluted by linear gradient with buffer B [50 mM KPi (pH 7.5); 500 mM NaCl; 0.01%w/v DDM; 0.5 mM EDTA]. Fractions containing cytochrome bc_1_ were pooled and concentrated to 0.5 mL using an Amicon Ultra-15 (Amicon, MWCO 100,000) concentrator. Concentrated sample was applied to a Sephacryl-S300 gel filtration column (ca. 120 mL) pre-equilibrated in buffer C [20 mM KMOPS (pH 7.2); 100 mM NaCl; 0.01%w/v DDM; 0.5 mM EDTA] and eluted at a flow rate of 0.5 mL/min. Purified cytochrome bc_1_ fractions were collected and concentrated to 40 mg/m. PEG fractionation with increasing concentration of PEG4000 was used to precipitate cytochrome *bc*_1_. Precipitating solution (100 mM KMES pH 6.4; 10% PEG4000; 0.5 mM EDTA) was mixed with the protein to a desired PEG concentration. The precipitated protein pellet was re-solubilised in buffer D (25 mM KPi pH 7.5, 100 mM NaCl, 0.5 mM EDTA, 0.015% DDM) and dialysed in the same buffer in a centrifugal ultrafilter to remove residual PEG. Five micromolars cytochrome *bc*_1_ was incubated at 4°C for 12 h with 50 μM JAC21 (10-fold molar excess) diluted from 20 mM solution stock in DMSO.

#### Crystallization, Data Collection, and Refinement of Cytochrome bc1–JAG21 Complex

The inhibitor-bound cytochrome *bc*_1_ was mixed with 1.6% HECAMEG to the final protein concentration of 40 mg/mL. Hanging drop method was used for crystallization. Two microliter of final protein solution with 2 μL of reservoir solution (50 mM KPi pH 6.8, 100 mM NaCl, 3 mM NaN_3_, 10–12% PEG4000) was equilibrated over reservoir solution at 4°C. The crystals were grown to 100 μm within 4 days. The single crystal was transferred in reservoir solution containing increasing to 50% concentrations of ethylene glycol prior to cryo-cooling in liquid nitrogen. X-ray data were collected from single crystal PROXIMA2 beamline, SOLEIL light source, France using DECTRIS EIGER X 9M detector at 0.9801Å wavelength up to 3.45Å resolution. Data were indexed and integrated using iMosflm (Battye et al., 2011), and scaled using Aimless (Evans, 2011). The starting model for refinement was 5OKD. All ligands except co-factors were removed from the model prior to refinement. Jelly-body refinement was carried out with Refmac5 (Murshudov et al., 2011). The inhibitor model was generated by Jligand (Lebedev et al., 2012). The model was manually edited in COOT (between cycle refinements. Data collection and refinement statistics are shown in Supplemental Table 1A).

### Cryo Electron Microscopy

#### Electron Microscopy and Image Processing

Cryo-EM was carried out as described in Amporndanai et al. (2018). Briefly, 3 μL of sample at 5 mg/mL concentration were applied to Quantifoil Cu R1.2/1.3, 300 mesh holey carbon grids and plunge frozen using an FEI Vitribot (blot time 6 s, blot force 6). Data were collected on an FEI Titan Krios with a Falcon III direct electron detector operated in integrating mode at 300 kV. Automated data collection was carried out using EPU software with a defocus range of −1 to −3.5 μm, and a magnification of 75,000 × which yielded a pixel size of 1.065 Å. Data were collected for 72 h resulting in 5,356 micrographs. The total dose was 66.4 e^−^/Å over a 1.5 s exposure which was split into 59 frames. All of the processing was performed in RELION 2.1 unless otherwise stated. The initial drift and CTF correction was carried out using MOTIONCORR2 (Zheng et al., 2017) and Gctf (Zhang et al., 2016), respectively. The micrographs were examined and those with crystalline ice were initially removed resulting in 2,960 micrographs. A subset of ~2,000 particles were manually picked to generate 2D references to facilitate auto-picking resulting in 439,009 particles. These particles underwent an initial round of 2D classification with those classes that displayed clear secondary structure detail being taken forward to 3D classification and split into three classes. Two of the three classes generated a high-quality cytochrome *bc*_1_ reconstruction with secondary structure information clearly visible. The particles from these two classes were recombined to form the final datasets consisting of 211,916 particles in the final reconstruction. The particles were 3D refined using C2 symmetry to produce a map with resolution 3.8 Å. The particles also underwent movie refinement and particle polishing which further improved the resolution of the map to 3.7 Å. A previously refined EM structure for SCR0911 (pdb 6FO6) was fit into the map using UCSF chimera and subsequently refined using phenix with the correct ligand. The maps were then inspected manually in COOT (Emsley and Cowtan, 2004) and the model corrected for any errors in refinement and the placement of residues.

### Statistical Analysis

A Pearson test was used to confirm a correlation between increasing dose and increasing inhibition. An ANOVA and subsequent pairwise comparison with Dunnett correction was used to determine whether or not inhibition or toxicity at a given concentration was statistically significant. Stata/SE 12.1 was used for this analysis.

## Results

### THQ Compounds Are Potent *in vitro*

**Figure 2:**
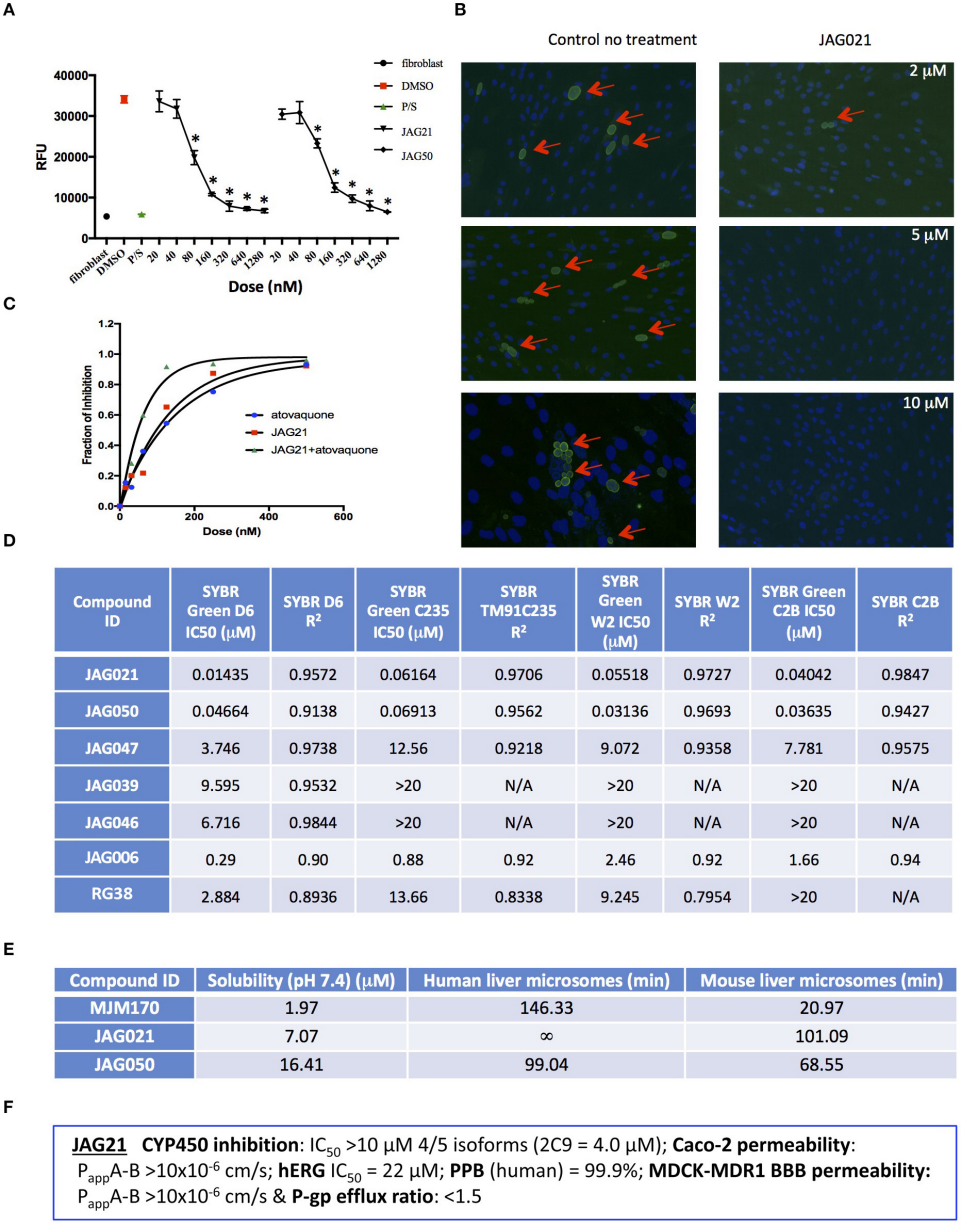
**JAG21** is potent *in vitro* against *Toxoplasma gondii*, tachyzoites and bradyzoites, and multiple drug resistant strains of *P. falciparum*. **(A) JAG21** is effective against RH-YFP tachyzoites, and does not harm human cells. Potent effect of JAG50 is also shown. A representative experiment is shown. *N* = triplicate wells in at least 2 biological replicate experiments. Relative fluorescence units are shown on the vertical axis, where decrease in fluorescence compared to diluent DMSO in media control indicates parasite inhibition (*p < 0.05). Horizontal axis indicates different treatment conditions: This shows results of testing of fibroblasts in media (HFF), DMS0 control, positive control pyrimethamine and sulfadiazine(P/S), and concentrations of JAG21 and JAG50 utilized. Differences were not statistically significant in the cytotoxicity assay (data not shown). **(B)** JAG21 is effective against EGS bradyzoites. Effect of JAG21 in reducing bradyzoites in HFF by parasite strain EGS. HFF were infected by EGS and treated with JAG21 at concentrations indicated. Slides were stained with Dolichos Biflorus Agglutinin conjugated with FITC (which stains the cyst wall) and DAPI, and observed with fluorescence microscopy. The red arrows point to the Dolichos enclosed organisms formed in tissue culture. These were eliminated with treatment with JAG21. This experiment was performed >4 times. These experiments were performed with 3 different observers reviewing slides at the microscope quantitating fields for each condition. Slides were also scanned and the scans of the slides were reviewed so all fields in the entire slide were noted to be consistent. **(C)** Synergy of JAG21 and atovaquone against Rh-YFP tachyzoites *in vitro*. Isobologram comparing JAG21, atovoquone, and JAG21 plus atovaquone demonstrates synergy. **(D)** THQs effective against drug resistant *P. falciparum*. Dose-response phenotypes of a panel of *P. falciparum* parasite lines. IC50 values were calculated using whole-cell SYBR Green assay and listed as mean ± standard deviation of three biological replicates, each with triplicate measurements. The D6 strain is a drug sensitive strain from Sierra Leone, the TM91-C235 strain is a multi-drug resistant strain from Thailand, the W2 strain is a chloroquine resistant strain from Thailand, and the C2B strain is a multi-drug resistant strain with resistance against atovaquone. **(E)** Solubility and Stability in human and mouse liver microsomes comparing MJM 170, JAG21, and JAG50. Performed by Chem Partners. **(F)** JAG21 CYP450 Inhibition, CACO-2, hERG, PPB, BBB (MDCK-MDK1) efflux analyses. These were performed by Chem Partners and are as defined in the section Materials and Methods. RG38 is a structurally related inactive THQ analog.

**Figure 3:**
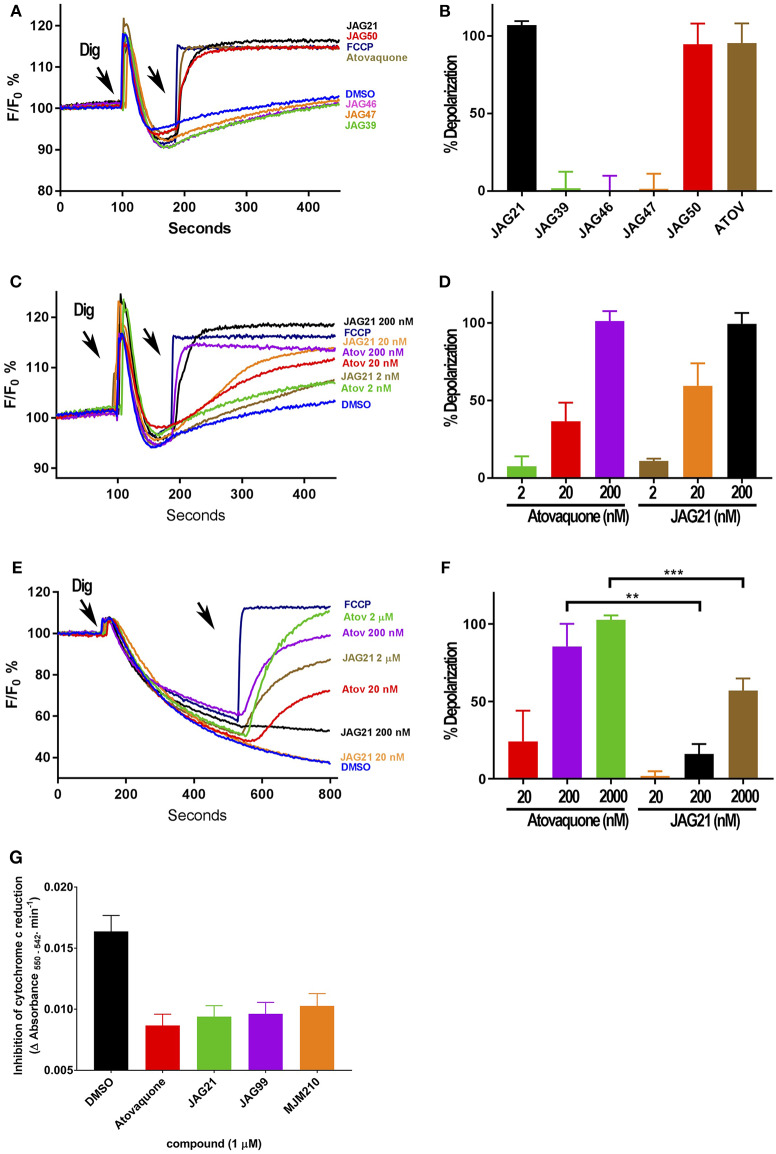
Effect of JAG21, and other THQ compounds on mitochondrial functions of *Toxoplasma gondii, Plasmodium falciparum* and HFF-hTE RT **(A)**. Maximum mitochondrial membrane depolarization of JAG21, JAG39, JAG46, JAG47, JAG50, and Atovaquone (4 μM) and FCCP (5 μM). Digitonin was added where indicated by the arrow to permeabilize cells and permit a necessary mitochondrial substrate (Succinate) to reach intracellular organelles. The addition of the indicated compounds is shown by the second arrow. **(B)** Quantification of the depolarization shown in **(A)**. The relative depolarization of each compound was normalized to the depolarization by FCCP which was considered 100% depolarization. **(C)** Effect of various concentrations of JAG21 and Atovaquone on the mitochondrial membrane potential measured as in **(A)**. The first arrow indicates digitonin addition and the second arrow indicates the addition of compounds at the specified concentration. **(D)** Quantification of the depolarization measured in **(C)**. The relative depolarization of each compound was normalized to the depolarization by FCCP (100%). **(E)** Mitochondrial membrane depolarization of HFF-hTERT in suspension by JAG21 and atovaquone. The first arrow indicates the addition of digitonin, and the second arrow indicates addition of the indicated compounds at the indicated concentration. **(F)** Quantification of the depolarization measured in **(E)**. The relative depolarization of each compound was normalized with the depolarization by FCCP, which was considered 100%. **(B,D,E)** X ± S.D., *N* = 3 independent experiments. Statistical analysis (unpaired student *t*-test) was performed using GraphPad Prism 8.0 (GraphPad Software, Inc., San Diego, CA). **P < 0.01. ***P < 0.001. **(G)** JAG21, JAG99, and MJM210 (1 μM) inhibited *P. falciparum* cytochrome c reduction. Vehicle (DMSO)/atovaquone (1 μM) were negative/positive controls, 1,290 respectively. X ± S.D., *N* = 4 independent experiments.

Initially, a small library of seven compounds (Figure 1 [blue and green font, Figure 1A] and Figure 2) were tested, and each compound was tested at least twice against *T. gondii* tachyzoites. JAG21 and JAG50 demonstrated effect below 1 μM, and were tested at lower concentrations. JAG50 and JAG21 were identified as lead compounds given the IC_50_ values obtained were 33 and 55 nM, respectively. Correlation between concentration of compound and inhibition of parasite replication (as measured by fluorescence) was observed for all compounds except JAG46. The relative effect on HFF and parasite enzymes were also compared, with those marked ^**^ in Figure 1A having the most effect on the parasite enzyme activity relative to host HFF enzyme activity as shown below in Figure 3.

A representative graph of these *in vitro* data is shown in Figure 2A. Subsequently, a larger library of 54 compounds was synthesized to ascertain structure-activity relationships (SAR) (Figure 1B). Our primary aims were to block putative metabolism of the terminal phenol ring of MJM170 and improve the solubility across the compound series. Substituents were generally tolerated at the meta and para positions on the phenol ring (R_**1**_), similar to the trends observed in the ELQ series (Vidigal et al., 2002; Doggett et al., 2012; McPhillie et al., 2016). The incorporation of heteroatoms into the aryl rings of the biphenyl moiety did not lead to improvements in solubility and biological activity. Small substituents were tolerated at the 7-position of the THQ bicyclic ring (Figure 1B; R_**1**_**)**, improving selectivity (see below, SAR) but not at the 6-position unlike the ELQ series. In summary, overall, nitrogen atoms were not tolerated in aryl ring (C) and the 4-position was optimal for phenol substituent. Ultimately, no other compound had all the advantages of JAG21, although some of these were identified as potential back up compounds (marked with ^**^), with greater selectivity for the parasite relative to the mammalian enzyme activity. Compound JAG21 displayed synergy against RH strain tachyzoites with atovaquone (Figure 2C) but not with pyrimethamine, although no antagonism was observed (data not shown).

Cytotoxicity assays performed in parallel using HFF, WST-1 (Fomovska et al., 2012a,b), and HEP G2 cells demonstrated a lack of toxicity at concentrations substantially in excess of the concentrations effective against tachyzoites. Because *T. gondii* grows inside cells, if a compound were toxic to host HFF then it would make the compound appear to be spuriously effective (Fomovska et al., 2012a,b), when in actuality only toxicity for the host cell would be measured. Cytotoxicity to HFF was therefore assessed for all compounds at 10 μM. Results of this experiment are in Figure 1A, toxicity column. A two-way ANOVA and subsequent pairwise comparison found none of the differences in absorbance, compared to the media-DMSO vehicle controls, to be statistically significant (*p* > 0.05). Most of these compounds are not toxic at 10 μM (the limit of solubility) and that cytotoxicity to cells can be attributed to DMSO in the solution, not the compound. Dose response testing (IC50) was performed with HEP G2 cells as described and the observed toxicity was: HEP G2 IC50 17.70 μM (*r*^2^ = 0.97) for JAG21; 7.1 μM (*r*^2^ = 0.98) for JAG50.

Lead compounds JAG50, JAG21, and others were tested against EGS strain (Paredes-Santos et al., 2013, 2018; Vidigal et al., 2002; McPhillie et al., 2016) tachyzoites and encysted bradyzoites using methods described earlier (McPhillie et al., 2016). We found a number of these compounds including JAG21 were highly effective against tachyzoites (RH-YFP; Fomovska et al., 2012a) (Figures 1A, 2A,C) and bradyzoites of EGS (Paredes-Santos et al., 2013; Vidigal et al., 2002; McPhillie et al., 2016; Paredes-Santos et al., 2018) (Figure 2B). For example, in a separate experiment (data not shown) using immunofluorescence microscopy, the following forms were observed: “true cysts” with a dolichos-staining wall, “pseudocysts” or tight clusters of parasites, and small organisms. If there were fewer than four parasites visible in a cluster, organisms were counted individually (as “small organisms”). A statistically significant reduction in the number of true cysts and small organisms was observed at 1 and 10 μM for both compounds (*p* < 0.05, *p* < 0.005). Five hundred nanomolars JAG21 treatment results in cultures where we do not see EGS bradyzoites (e.g., Figure 2B).

Results against *P. falciparum* using methodology described earlier (Trager and Jensen, 2005; Johnson et al., 2007; Plouffe et al., 2008; McPhillie et al., 2016) also are shown in Figure 2D. JAG 21 is potent against *P. falciparum* with IC50 values ranging from 14 to 61 nM against a variety of drug sensitive and resistant strains (McPhillie et al., 2016) including D6, TM91-C235, W2, and C2B. The D6 strain is a drug sensitive strain from Sierra Leone, the TM91-C235 strain is a multi-drug resistant strain from Thailand, the W2 strain is a chloroquine resistant strain from Thailand, and the C2B strain is a multi-drug resistant strain resistant to atovaquone. Effects of other comparison compounds are also shown in this table and range from 31 to 20,000 nM (Figure 2D).

### ADMET Superiority of JAG21

*In vitro* absorption, distribution, metabolism, excretion, and toxicity (ADMET) analyses of the THQ compounds were outsourced to ChemPartner Shanghai Ltd. ELQ-271 (synthesized in-house) was tested as a comparison. THQs which were potent inhibitors of *T. gondii* tachyzoites were assessed for their kinetic solubility, metabolic stability in human, and mouse liver microsomes (Figure 2E), hERG, and their ability to permeate across MDCK-MDR1 cell membranes (*in vitro* measure of blood-brain barrier (BBB) penetration potential/permeability). Solubility, half-life, HERG, and BBB permeability/efflux results are shown in Figure 2F. The aqueous solubility (PBS, pH 7.4) of amorphous compounds JAG21 and JAG50 was 7 and 16 μM, respectively, which is improved over MJM170 (2 μM) and ELQ-271 (0.2 μM). We also tested solubility of the microcrystalline form of JAG21 and found that the solubility was 3.5 μM. JAG21 was the most metabolically stable compound in human liver microsomes (>99% remaining after 45 min) compared with other THQs and ELQ-271, although it displayed a much shorter half-life of 101 min in mouse liver microsomes. All THQs tested in the MDCK-MDR1 system for blood brain barrier (BBB) permeability (including MJM170, JAG21, and JAG50), exhibited high permeability (P_app_ >10 × 10^6^ cm/s) and low efflux (efflux ratio <1.5).

### THQs Potently Inhibit Parasite Cytochrome bc1 (Cytbc1) Enzyme Activity

JAG21 is the most active of the initially tested THQs against *T. gondii* Cytbc1, which also showed selectivity for the parasite over the mammalian mitochondrial membrane potential (Figure 3). Following the full SAR testing *in vitro* against tachyzoites, the full set of compounds was tested against HFF; then the initial compounds also were tested against the *T. gondii* and HFF enzyme benchmarked against atovaquone, and ultimately the full set of compounds was compared for effect against the *T. gondii* and HFF enzymes.

Mitochondrial membrane potential measurements were performed with permeabilized *T. gondii* tachyzoites in suspension using safranin O, which loads into polarized membranes [see section Materials and Methods in the Supplemental Materials (Vercesi et al., 1998)]. *T. gondii* tachyzoites were permeabilized with digitonin to allow the mitochondrial substrate succinate to cross the membrane and energize the mitochondrion. The fluorescence of safranin O, which loads into energized mitochondria was used to measure the membrane potential. The energized state of the mitochondrion is observed by a decrease in fluorescence (Figures 3A,C,E). Trifluoromethoxy carbonylcyanide phenylhydrazone (FCCP) was used to depolarize the membrane, which is observed as an increase in fluorescence (Figures 3A,B). JAG21 depolarized the membrane potential even at concentrations as low as 2 nM (Figures 3C,D). JAG21 and Atovaquone had similar effects on the mitochondrial membrane potential (Figure 3D). Other compounds like JAG46 and 47 showed almost no effect at doses as high as 4 μM (Figures 3A,B). JAG50 showed depolarizing activity at doses of 200 nM and higher. The effect of these THQ compounds against the *T. gondii* mitochondrial membrane potential was greater than the effect on the human foreskin fibroblast mitochondrial membrane potential (Figures 3E,F). This is consistent with the observation that JAG21 is less toxic against human Telomerase reverse transcriptase immortalized (hTERT) HFF cells than atovaquone. We had newly created THQ compounds, not yet characterized fully, that show even less toxicity to the human fibroblast cytochrome b/c complex marked with ^**^ in Figure 1A. These could be developed in a second phase of our program, were reductions in toxicity needed. However, as data presented herein demonstrates, there are significant advantages in the ADMET properties of JAG21, and its dramatic efficacy *in vivo*, without toxicity. There may be no need to further develop any of those potential additional leads.

Enzyme reduction of cytochrome *c* by *P. falciparum* parasite extract (Fisher et al., 2004, 2009) is mediated by *P. falciparum bc*_1_ complex cytochrome *c* reductase (*Pfbc*_1_) enzyme. All three compounds tested (1 μM) significantly inhibited the reduction of cytochrome *c* by the *P. falciparum* parasite extract (JAG21 = 86.4 ± 3.2; JAG99 = 81.3 ± 6.0; MJM170 = 69.7 ± 11.3% of the atovaquone response, Figure 3G. Additional data demonstrated selective effect on *P. falciparum* enzyme compared with bovine enzyme (data not shown).

### Binding, Co-crystallography, Pharmacophore, and Cryo-electron Microscopy Studies Demonstrate Selectivity

**Figure 4:**
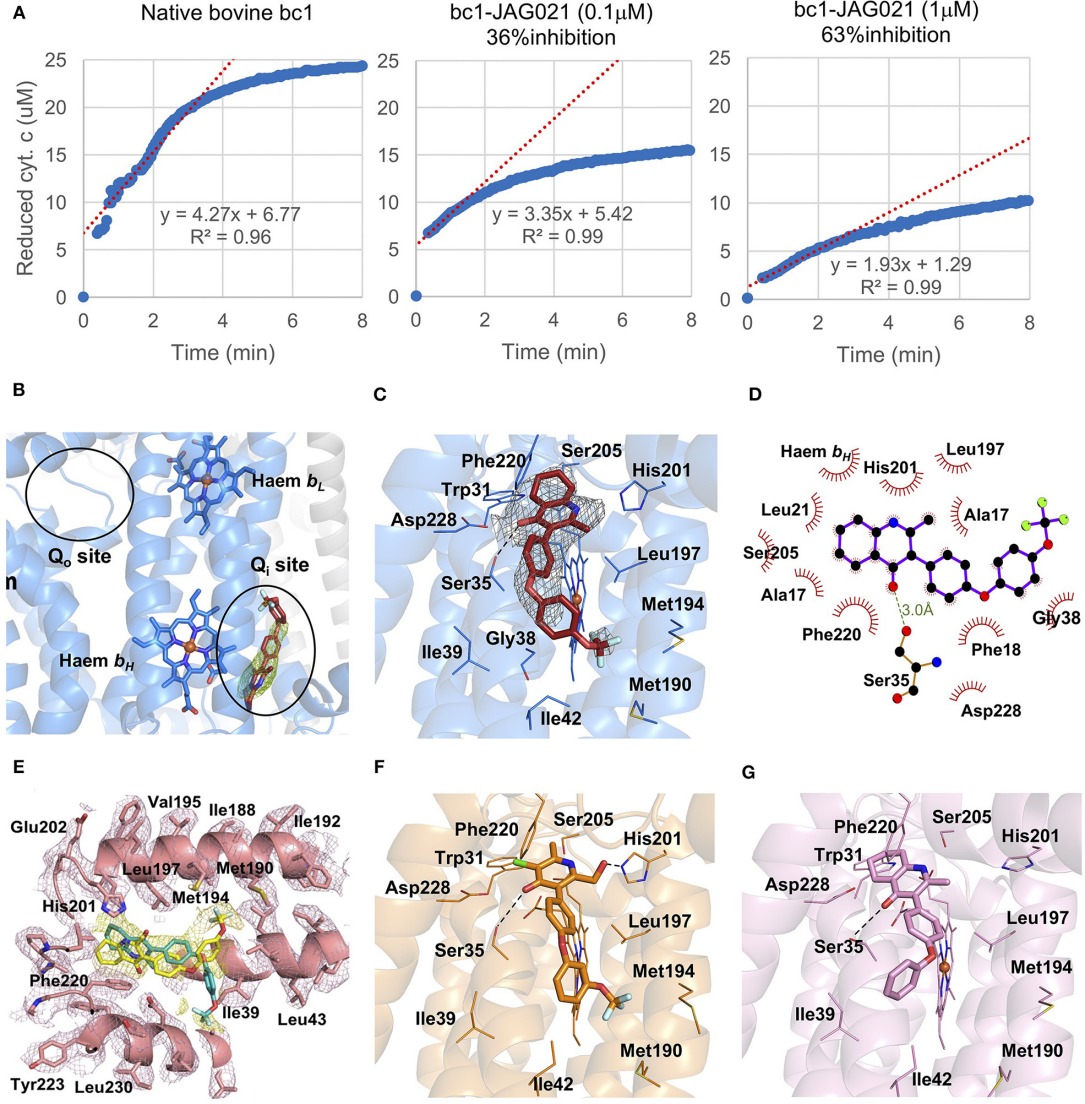
Binding studies of JAG21 to bovine bc1. **(A)** Bovine Cytbc1 activity assays showing 36 and 63% inhibition at 0.1 and 1 μM concentration of JAG21, respectively. *N* = at least 2 biological replicate experiments with similar results. **(B)** The Cytbc1 structure presented in cartoon style with clear omit (Fo-Fc) electron density map for the bound JAG21 compound only in the Q_i_ site showing selectivity within the binding pocket. Qi and Qo sites are marked by black ellipsoids. **(C)** The bound JAG21 compound (orange) within the Qi site with corresponding (2Fo-Fc) electron density map contoured at 1 σ level as gray mesh. The residues which make close interactions with the bound inhibitor are shown in stick format and labeled. **(D)** 2D pharmacophore analysis of JAG21 binding pocket produced using Ligplot+ LS-2011. Hydrophobic interactions are shown as red spikes, hydrogen bond with Ser35 is shown by green dashes. **(E)** Cryo-EM derived structure of the Cytbc1 bound JAG021 structure with corresponding density map contoured at 3 σ level suggesting two different positions for the head group represented by two regions of density shown as yellow mesh. The Cytbc1 structure bound to the pyridone GSK932121 (PDB:4D6U) **(F)** and quinolone MJM170 (PDB:5NMI) **(G)** in the Q_i_ site. Haem and compounds are shown as colored sticks, Fe ion as orange sphere and hydrogen bonding as black lines. Hydrogen bonding with Ser35 is shown as black dashes. Terms JAG021 and JAG21 used interchangeably for this same compound.

In binding assays and in co-crystallography (Emsley and Cowtan, 2004; Emsley et al., 2010; Battye et al., 2011; Laskowski and Swindells, 2011; Murshudov et al., 2011; Lebedev et al., 2012; Capper et al., 2015; McPhillie et al., 2016; Zhang et al., 2016; Zheng et al., 2017; Amporndanai et al., 2018), JAG21 has lower binding affinity to bovine cytochrome *bc* in comparison with previous compounds that we have tested. JAG21 “inhibits” Cyt*bc1* but not fully, indicating that it will be less toxic for mammalian (bovine/human) cyt *bc1* than the apicomplexan enzymes (Figure 4A). The electron density map in the Qi site of bovine cytochrome *bc*_1_complex with JAG21 (Supplemental Table 1, Data Collection Statistics) reveals an additional electron density, which allowed unambiguous positioning of the inhibitor (Figure 4B). No additional electron density was found within the Q_o_ site. After the refinement, 2F_o_-F_c_ electron around JAG21 becomes clearer (Figure 4C). The second aromatic ring in the tail group of the compound is less defined due to high flexibility introduced by the oxygen linker. The quinolone head of JAG21 is held between Asp228 and His201 and adapted the same conformation as 4(1H)-pyridone (GSK932121) (Capper et al., 2015) (Figure 4D) and tetrahydro-4(1H)-quinolone (MJM170) (McPhillie et al., 2016) (Figure 4E) by directing the NH group to His201 and the carbonyl group to Asp228. The carbonyl of the quinolone head and OG1 atom of Ser35 are within 3.0 Å distance that allows hydrogen bonding and enhances the binding affinity to the bovine enzyme. The 3-diarylether tail extends along a hydrophobic channel defined by Gly38, Ile39, and Ile42. The trifluoromethoxy group at the phenoxy ring points toward Met190 and Met194 (Figure 4F). CryoEM studies of the complex also demonstrate reasons for selectivity. In Figure 4F, the density suggests that the inhibitor can adopt two different binding poses as observed previously in the cryo-EM structure of GSK932121 (Capper et al., 2015). The binding pose shown in yellow, which has the strongest density, agrees with the crystal structure and has the trifluoromethoxy group pointing toward Met194. However, there is additional density which could result from a second binding pose (green) in which the trifluoromethoxy group points toward Asp228 (McPhillie et al., 2016). Figure 4F shows GSK932121 pyridone (PDB:4D6U) (Figure 4G) MJM170 quinolone (PDB:5NMI). The EM map has been deposited at the EMDB (EMDB-11002).

### JAG21 Is Potent *in vivo*

**Figure 5:**
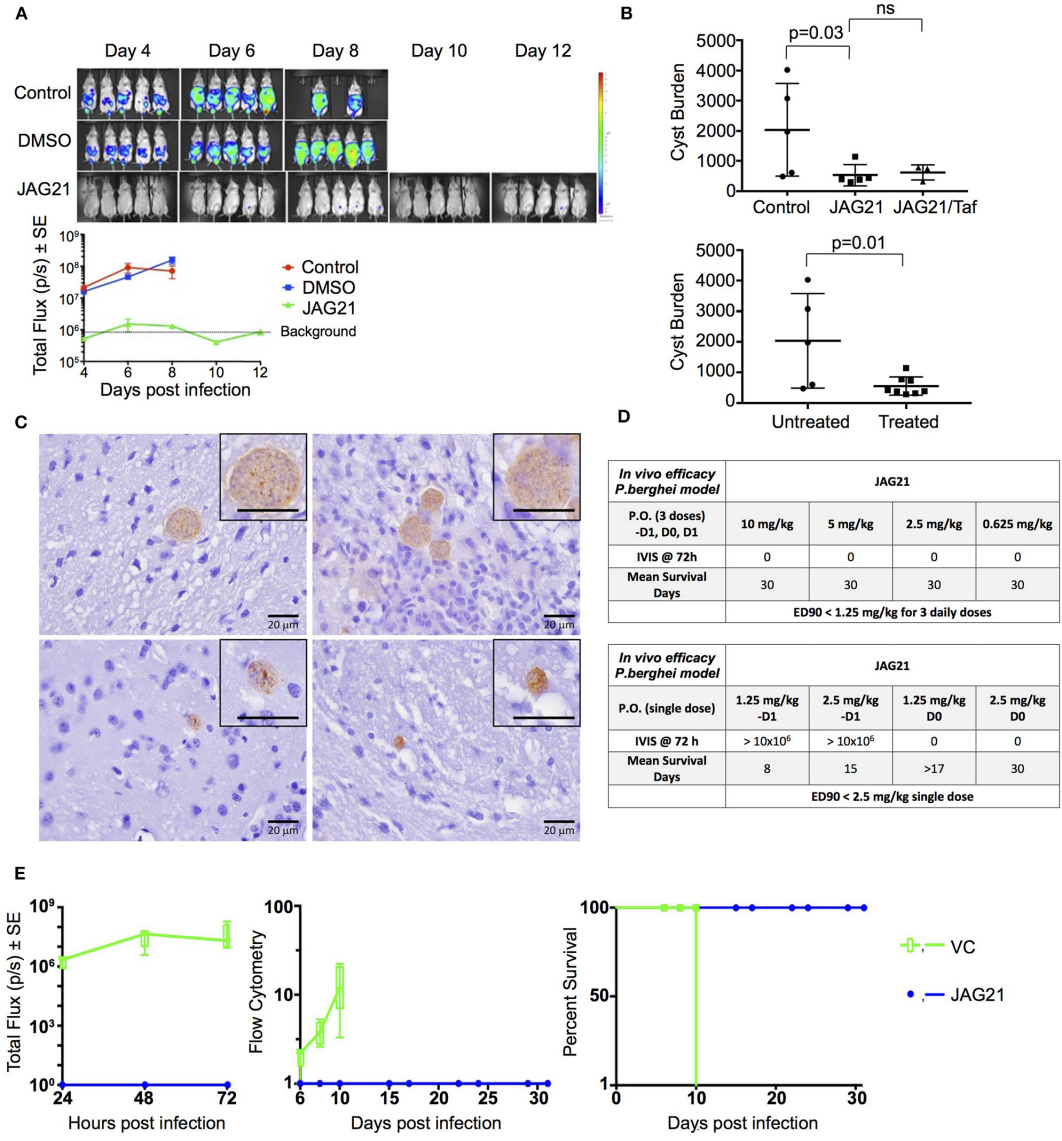
JAG21 is a mature lead that protects against *Toxoplasma gondii* and *Plasmodium berghei in vivo*. **(A)** JAG21 treatment for 14 days protects against *T. gondii* tachyzoites *in vivo*. Tachyzoite challenge with Prugneaud luciferase parasites imaged with leuciferin using IVIS demonstrates that treatment with JAG21 eliminates leuciferase expressing parasites and leads to 100% survival of JAG21 treated infected mice. No cysts were found in brains of mice at 30 days after infection when they have been treated with JAG21 for the first 14 days after infection. There were 2 biological replicate experiments with 5 mice per group with similar results. **(B)** JAG21 and JAG21 plus tafenoquine markedly reduce Me49 strain brain cyst numbers *in vivo* in Balb/C mice at 30 days after infection. Parasites were quantitated by scanning the entire immunoperoxidase stained slide in an automated manner and by two observers blinded to the experimental treatment using microscopic evaluation. In each of two experiments, the numbers of mice per group were as follows: Experiment 1 had 4 diluent controls, 5 JAG21, 4 JAG21/Tafenoquine treated mice; and Experiment 2 had 5 diluent controls, 5 JAG21, 3 JAG21/Tafenoquine treated mice. Immunoperoxidase staining was performed. Parasite burden was quantitated using a positive pixel count algorithm of Aperio ImageScope software. Positive pixels were normalized to tissue area (mm^2^). Quantification was by counting positive pixels per square area. The entire brain in one section was scanned for each mouse. The parasite burden was quantitated as units of positive pixels per mm^2^. The average ± S.E.M. numbers of mm^2^ per slide quantitated was 30.2±1.6 mm^2^ per mouse for this quantification. Each high power field of view shown in **C** is ~0.02 mm^2^ per field of view. A representative single experiment is presented and the data from the two experiments analyzed together also demonstrated significant differences between the untreated and treated groups (*p* < 0.01; Supplemental Figure 1). **(C)** Microscopic evaluation of the slides reveal effect of JAG21 and JAG21 plus tafenoquine having the same pattern as the automated quantitation of immunoperoxidase stained material. There are usual appearing cysts in the DMSO control untreated mice as shown in the top panels, and rare cysts in the treated mice with most of the brown material appearing amorphous (bottom panels). **(D)** JAG21 nanoformulation dosages administered to *P. berghei* infected C57Bl6/albino mice compared with vehicle control. Design of single dose and 3 day dose experiments. **(E)** JAG21 nanoformulation cures *P. berghei* sporozoites (left panel), blood (middle panel), and liver stages, leading to 100% survival (right panel). This is with oral administration of a single dose of 2.5 mg/kg or 3 doses at 0.625 mg/kg. Single dose causal prophylaxis in 5 C57BL/6 albino mice at 2.5 mpk dosed on day 0, 1 h after intravenous administration of 10,000 *P. berghei* sporozoites. Shown is 3 dose causal prophylaxis treatment in 5 C57BL/6 albino mice at 0.625 mpk dosed on days −1, 0, and +1. Representative figure showing survival (right panel), luminescence (left panel), and parasitemia quantitated by flow cytometry (middle panel) for 5 mg/kg.

*In vivo* studies of JAG21 against *T. gondii* demonstrated high efficacy in a variety of settings. JAG21 at 5 mg/kg/day administered IP improves well-being and eliminates illness and *T. gondii* Type II Prugneaud luciferase tachyzoites completely in luminescence studies (Figure 5A). Further, treatment beginning on day one after infection results in no cysts being found in brains of these mice treated for 14 days with 5 mg/kg/day of JAG21, when brains were evaluated 30 days after stopping JAG21 treatment in two replicate experiments. Treatment beginning on day 30 after initiation of infection with Type II Me49 parasites results in marked, statistically significant reduction in normal appearing cysts, free organisms, and immunoperoxidase stained cysts detected by automated imaging of scanned slides (Figures 5B,C, *p* < 0.03 experiment 1: *p* < 0.01 experiments 1 and 2 together, Supplemental Figure 1). The automated analysis confirmed results from the blinded microscopic visual quantitation of cysts and free organisms in slides by two observers. Adding tafenoquine or primaquine to treatments of active plus dormant malarias (St Jean et al., 2016; Lacerda et al., 2019; Llanos-Cuentas et al., 2019) is partially effective against both active and dormant phase plasmodia, when neither treatment of active nor dormant disease alone is effective for either *in vivo*. We developed experiments based on these observations where experiments with tafenoquine alone or with JAG21 alone was used in the experiments with established cysts with immune competent mice. This was to determine whether tafenoquine might add to efficacy of JAG21. The efficacy of treatment with JAG21 alone was so robust (Figure 5B), that no additive effect was seen, or could have been detected, by adding Tafenoquine to JAG21. Efficacy was shown when data were analyzed as separate groups, i.e., control vs. JAG21 alone (*p* < 0.03) or control vs. JAG21 plus tafenoquine, or grouping the JAG21 and JAG21 plus tafenoquine results as “untreated” vs. “treated” (*p* < 0.01). Analysis shown combining both treatment groups from two replicate experiments showed similar results (*p* < 0.01, Figure 5B), and when results from replicate experiments were grouped (Supplemental Figure 1). In Figure 5C the control mice had cysts with usual morphology (Top two panels), whereas treated mice had very few morphologically recognizable usual cysts that were immunostained (bottom panels).

A nano formulation homogenized (<6 μM) was used effectively orally for the *P. bergheii* experiments, further, importantly, was effective in the single oral dose causal prophylaxis in 5 C57BL/6 albino mice at 2.5 mg/kg dosed on day 0, 1 h after intravenous administration of 10,000 *P. berghei* sporozoites was completely protective. In addition, 3 dose causal prophylaxis treatment in 5 C57BL/6 albino mice at 0.625 mg/kg dosed on days −1, 0, and +1 also was completely protective. A representative experiment at a higher dose (5 mg/kg) is shown, but all experiments with the oral dosing regimen with the nanoformulation specified above showed 100% survival 30 days post infection with *P. berghei*, where all liver and blood stage parasites were eliminated (Figures 5D,E) demonstrates not only efficacy of JAG21 against the three life cycle stages of *P. berghei*, but also demonstrates the efficacy of oral administration of the nanoformulation when used immediately, at a low dose.

### G1 Arrest, Persisters, Companion Compounds

**Figure 6:**
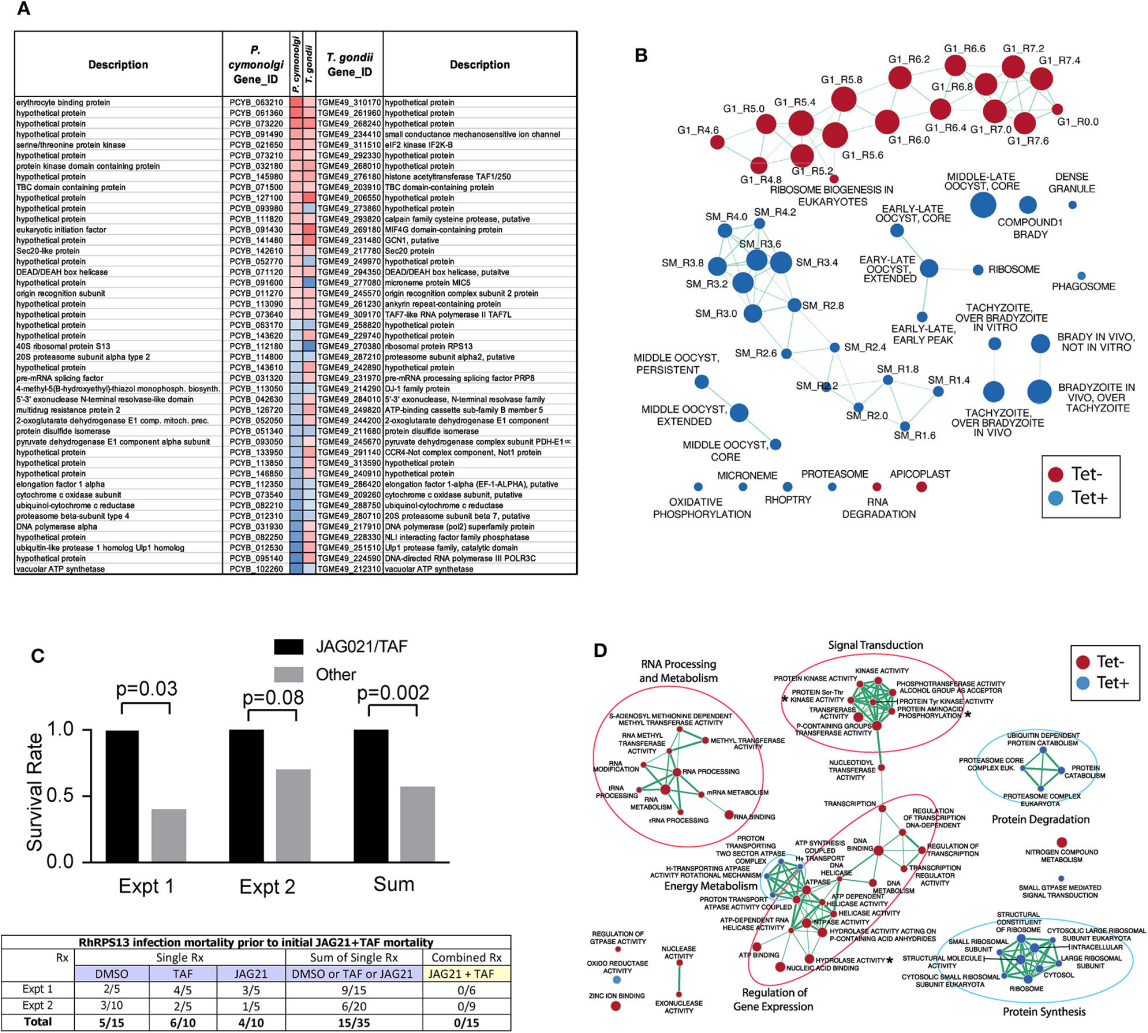
*Toxoplasma gondii* ΔRPS13 transcriptome during Primary Human Brain Neuronal Stem Cell (NSC) infection and *in-vivo* susceptibility to JAG21 and TAF treatment are reminiscent of literature findings with malaria hypnozoites. **(A)** *P. cynomolgi-T. gondii* best reciprocal match genes significantly upregulated (red) or downregulated (blue) in *P. cynomolgi* hypnozoites compared to liver-schizont stage and in ΔRPS13 after downregulation of *rps13* gene expression (*p* ≤ 0.05, FDR ≤ 0.2). **(B)** Gene-set enrichment analysis of ΔRPS13 +/– Tc. Blue and red nodes denote gene-sets enriched in presence or absence of Tc, respectively. Node diameters are proportional to number of genes belonging to corresponding gene-sets. Edge thickness is proportional to number of genes shared between connected nodes. **(C)** Survival rate of mice infected with 100,000 ΔRPS13 followed by treatment with diluent, JAG21, tafenoquine (TAF) or the two together (JAG21/TAF). Then tetracycline was added at 14 days. The combination of the two compounds resulted in improved time of survival (*p* < 0.03, Experiment 1; *p* = 0.08 Experiment 2, *p* = 0.002 Experiment 1+2). The full data are presented in the box below the image in **(C). (C)** Rx refers to treatment of mice with diluent (DMSO), Tafenoquine (TAF), or JAG21, or JAG21 and TAF. ΔRPS13 is referred to as RhRPS13Δ in the title of the box in **(C)**. In Supplemental Figure 2, histological preparations that are immunoperoxidase stained for *T. gondii* antigens from a pilot study were prepared (Supplemental Figure 2). These are images, in Supplemental Figure 2 of liver and spleen from IFN γ receptor knock out mice without treatment on days 7 and 14 after infection. In those mice without any treatment there was amorphous brown immunoperoxidase stained material in Supplemental Figure 2A. When tetracycline (aTet) was administered on day 14 after infection in drinking water, with tissues obtained and immunostained for *T. gondii* antigens from mice that died or became very ill, organisms that were clearly recognizable could be seen (Supplemental Figures 2B–E). Design of the treatment experiment with control DMSO diluent, JAG21 alone, Tafenoquine alone (TAF) or the two together (JAG/TAF) with full data for each of the groups and with the composite analysis from replicate experiments, including numbers of mice, are shown in Supplemental Figures 2C,D. Supplemental Figures 2C,D shows prolongation of survival time, but there is not durable protection against ΔRPS13 in these immune compromised mice treated with JAG21/TAF as described. This is summarized in **C** to demonstrate early prolongation of survival time with the detailed data in Supplemental Figure 2. (D) Gene ontology enrichment analysis of ΔRPS13 +/–Tc. Node and edge conventions are the same as in **(B)**. There were at least 2 biologic replicates of each experiment.

In mice that were treated with JAG21 early after infection (Figure 5A) we could find no residual immunostaining for *T. gondii* in brain tissue of any mice. This suggests that very early treatment could prevent established, chronic infection, for example in epidemics such as those that occurred in Victoria, Canada, the U.S.A., and Brazil. In mice with established cysts, following treatment with JAG21, we occasionally saw a small number of cysts (Figure 5B) and amorphous immunostained structures (Figure 5C, bottom panels). This was reminiscent of persistence in some malaria infections (Cubi et al., 2017) and abnormal immunostained structures we previously identified with a conditional, tetracycline-on regulatable, mutant *T. gondii* (Hutson et al., 2010 and Supplemental Figure 2). In this ΔRPS*13* tachyzoite, small ribosomal protein 13 can be regulated, depending on whether anhydrotetracycline (ATc) is absent or present, leading the ATc responsive repressor to be on or off response elements engineered into the promoter (Hutson et al., 2010). ΔRPS*13* replicates with ATc present and is arrested in G1 when ATc is absent in HFF cultures (Hutson et al., 2010). The dormant parasite could persist for extended periods (Hutson et al., 2010). The parasite could be rescued from its dormant—ATc state by adding ATc, months after removing tetracycline from infected HFF cultures, although it could not be rescued in immunocompetent mice with LNAME and ATc when tested 1 week after infection (Hutson et al., 2010). We wondered if this type of dormant organism could form *in vivo*, whether it could contribute in a biologically relevant way to dormancy and recrudescence, similar to the malaria hypnozoite (Cubi et al., 2017; Muller et al., 2019), and whether JAG21 might be able to eliminate it, or whether a companion compound effective against this form might be needed or work in conjunction with JAG21 if needed. To begin to address these questions and to investigate how close the *T. gondii* ΔRPS*13*-ATc phenotype might be to the malaria hypnozoite, we compared the transcriptome of *T. gondii* ΔRPS*13* in human, primary, brain, neuronal stem cells +/– ATc to the recently published *P. cynomolgi* hypnozoite transcriptome, established with single cell RNA sequencing in laser captured organisms (Cubi et al., 2017). This analysis identified 28 orthologous genes with similar expression pattern in both *T. gondii* ΔRPS*13*-ATc and *P. cynomolgi* hypnozoites, including the downregulation of rps*13* and upregulation of the eukaryotic initiation factor-2α kinase IF2K-B, a protein involved in translational control in response to stress (Cubi et al., 2017) (Figure 6A). Further. assessment of the *T. gondii* ΔRPS*13* transcriptome in the absence or presence of ATc showed upregulation of additional IF2K members, 25 Apetela (AP) 2 transcription factors and a number of genes that participate as protein ubiquitin ligases, and in trafficking as well as in RNA binding, and GCN1 (Supplemental Table 2). None of them, except for AP2VIIa-7, have been shown to be upregulated nor downregulated during differentiation to bradyzoites. Gene set enrichment analysis showed that in the absence of ATc, the *T. gondii* ΔRPS*13* transcriptome is enriched in genes typically expressed during G1, confirming previous results indicating that downregulation of *rps13* arrests the parasite at this stage of the cell cycle (Figure 6B) (Hutson et al., 2010). Moreover, a number of biological processes are downregulated without ATc, including protein synthesis and degradation as well as energy metabolism (Figure 6B). Noteworthy, some gene ontology (GO) terms enriched in *T. gondii* ΔRPS*13*-Tc are also overrepresented in the *P. cynomolgi* hypnozoite (stars in Figure 6D). Further, without ATc the transcriptome of *T. gondii* ΔRPS*13* is compatible with a parasite transitioning from an active replicating form to a dormant stage, reflected by the downregulation of genes typical of the S and M stages of the cell cycle, and of genes that participate in energy metabolism and virulence (Figures 6B,D, Supplemental Table 2, Supplemental Figure 2). It has been reported that with treatment of active forms of malaria, hypnozoites still persist, and recrudesce later (Hutson et al., 2010; St Jean et al., 2016; Cubi et al., 2017; Lacerda et al., 2019; Llanos-Cuentas et al., 2019). Also, compounds that target cytochrome b/c were not effective against malaria hypnozoites. If primaquine or tafenoquine, which do not treat the active *Plasmodium vivax* parasites, were added *in vivo*, hypnozoites have been shown not to recrudesce, or do so less often (St Jean et al., 2016; Lacerda et al., 2019; Llanos-Cuentas et al., 2019). Testing with primaquine or tafenoquine could only be performed *in vivo*, as activity against the hypnozoite requires hepatic metabolism of primaquine or tafenoquine (St Jean et al., 2016; Lacerda et al., 2019; Llanos-Cuentas et al., 2019). Tafenoquine is not active in tissue culture which is consistent with the findings that these compounds require hepatic metabolism. To establish a parallel *in vivo* system, we studied immune compromised mice (Interferon ⋎ receptor knockout mice with the knockout in the germline) infected with ΔRPS*13* herein. Although in immune competent mice ΔRPS*13* does not recrudesce with ATc treatment initially, beyond 3 days after infection, we found that when ATc was added after treatment of the immune compromised mice with JAG21 dosed intraperitoneally for 14 days, the dormant ΔRPS*13* parasite could still recrudesce after JAG21 treatment was discontinued and tetracycline added (Figure 6C and Supplemental Figure 2). Consistent with adding tafenoquine to treatment of *P.vivax* malaria with chloroquine where both medicines together were partially effective against the active and hypnozoite forms, the combination of JAG21 and tafenoquine had a modest effect together on transiently improving survival time when ATc was added when compared with JAG21 or tafenoquine alone (Figure 6C and Supplemental Figure 2). The trend in the result seems similar to the malaria infections where hypnozoites form, although protection was not as robust, as in the malaria model, and we did not achieve complete, durable protection against ΔRPS*13*. These results in Figure 6C and Supplemental Figure 2 suggest: (a) In G1 arrested organisms that begin as tachyzoites, they can persist *in vivo* even if their morphology as parasites is difficult to discern; (b) Treatment with JAG21+Tafenoquine can prolong time to death more robustly than other treatments; (c) But, in these immune compromised mice at this dosage regimen this treatment did not robustly, durably protect these mice from death later; (d) In these immune compromised mice, whether this lack of complete protection was because of immune compromise, or less than optimal duration of treatment, or suboptimal dose or timing of treatments, or that this G1 arrested organism is harder to treat, remains to be determined in future studies. The modest efficacy of the two compounds, administered together, suggests that treating both tachyzoites and the G1 arrested organisms is important. This seems similar to *P. cynomogli* and *P. vivax* treatment with tafenoquine and chloroquine studies, which also showed efficacy but was not completely successful in preventing relapse. At the time this study was performed, formulation and dosing (including duration and timing) had not yet been optimized formally for the *T. gondii* model. *P. vivax* treatment requires chloroquine to treat blood schizonts and tafenoquine to treat hypnozoites. Treatment in man, per the FDA approved label, consists of a single dose of 300 mg on day 1 co-administered with chloroquine treatment on days 1 or 2. Both medicines have long half-lives in humans. This treatment was relatively effective in humans, with about a 30% recurrence rate.

Sinai et al. have demonstrated heterogeneity in the phenotypes of organisms within established cysts. Their work found bradyzoites within cysts are not uniform with regard to their replication potential (Watts et al., 2015), mitochondrial activity (Sinai, unpublished), and levels of the glucose storage polymer amylopectin (Sinai, unpublished). These properties of bradyzoites (Watts et al., 2015), and properties of tissue cysts that vary during the course of infection, demonstrated that there are unappreciated levels of complexity in the progression of chronic toxoplasmosis (Watts et al., 2015). The analysis (Figure 6D) of the ΔRPS*13* infected NSC suggests molecular targets that are modified in this G1 arrested ΔRPS*13* parasite as shown in Figure 6D and Supplemental Table 2. In the future, with formulation and pharmacokinetics of JAG21 optimized, it will be of interest to determine whether JAG21 can eliminate these organisms and any residual structures as in Figure 6C, or whether adding synergistic compounds such as atovaquone (Figure 4B) or antisense effective against these upregulated molecular targets, such as kinases, ATPases, AP2s (Figure 6D and Supplemental Table 2), or a newly recognized bradyzoite master regulator of differentiation might be effective alone or might be synergistic with JAG21 against this ΔRPS*13*, as well as the conventional recognized tachyzoite and bradyzoite life cycle stages. Chen et al. reported in the transcriptomes of established bradyzoite *in vivo* cysts that EIF2kinase of stressed parasites is present (Chen et al., 2018), but we have not found other overlap of Chen's transciptome with *P cynomogli* or ΔRPS*13* transcriptomes. Others have described EIF2kinase and stress granules only in transitioning or extracellular parasites (Watts et al., 2015). Bradyzoites within tissue cysts are not monolithic. Thus, in future studies, single cell RNA sequencing of bradyzoites obtained by laser capture of bradyzoites *in vivo*, defined on the basis of their physiological state, may be needed to determine whether a transcriptome signature similar to ΔRPS*13* is sometimes present. This could be linked to morphologic/immunostaining features that might functionally distinguish them to define the character of a hypnozoite-like state in *T. gondii*. We noted heterogeneity of parasite phenotype, even in the same vacuoles, in our earlier IFA and electron microscopic characterization of G1 arrested ΔRPS*13* in HFF (Hutson et al., 2010). Heterogeneity also was found very recently in tachyzoites and bradyzoites created by alkaline conditions in culture across the cell cycle *in vitro* in HFF, using single cell RNA sequencing (Xue et al. Biorx, 6/3/2019 in press). These authors also noted that what had been interpreted as “noise” earlier was found actually to be signal in a more complex environment. These authors suggest that such heterogeneity might make developing curative treatments more complex. Our analysis of JAG21 effects and the ΔRPS*13*-ATet knockdown herein begin to help address this question: We noted that consistent with heterogeneity in our IFAs, in our comparison with the Xue et al.'s heterogeneous P1-6 clusters analysis, we found that most of the up- or down-regulated genes are within P3-P5 tachyzoite clusters. Also, consistent with the heterogeneity we observed in our G1 arrested ΔRPS*13*-ATet comparison, ΔRPS*13* has a drop in SAG1 and elevated SRS44 that is consistent with a brady-like phenotype. BAG1 expression was too low overall to draw any conclusion about BAG1. It is also noteworthy that in our -ATet relative to +Atet conditions in primary, human, brain, neuronal stem cells, the master regulator of bradyzoite differentiation is slightly overexpressed (Log_2_ Fold Change=0.7, adjusted *p* = 0.043). Although JAG21 is highly potent against tachyzoites and bradyzoites, it did not eliminate every long-established encysted bradyzoite or -ATet ΔRPS*13* completely either *in vitro* or in IFNγ knock-out mice *in vivo*. Consistent with heterogeneity, herein JAG21 treatment of ΔRPS*13* and transcriptomics analyses define a metabolically quiescent, persister, “stasis” state that is reversible even after substantial periods of dormancy. These observations contribute to conceptual and functional understanding of both Plasmodia species and *T. gondii* infections and molecular mechanisms whereby “persisters” might be eliminated.

### An Oral Nanoformulation Is Potent Against Virulent RH

**Figure 7:**
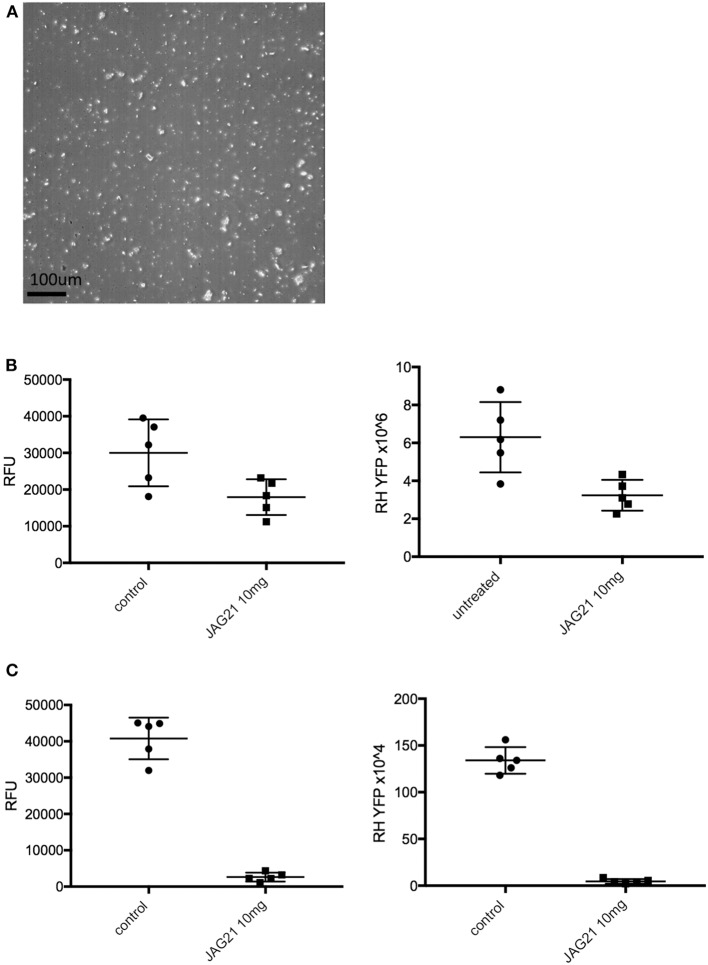
Oral nanoformulation of JAG21 potently protects against 2000 highly virulent RH strain tachyzoites given intraperitoneally. **(A)** Following sonication produces nanoparticles of ~2.86 μM. **(B)** Single oral dose of 10 mg/kg reduced intraperitoneal tachyzoites measured by RH YFP expression and counting with hematocytometer (*p* < 0.03). **(C)** Three daily 10 mg/kg doses markedly and significantly reduces intraperitoneal parasite burden measured as fluorescence and by hematocytometer on the fifth day (*p* < 0.001). No compound was administered after the third day. *N* = at least 2 biological replicate experiments with 5 mice per group with similar results.

To further develop JAG21 for practical, clinical use, our next step was to make a formulation that is stable at room temperature, and would be effective when administered orally: Following a number of unsuccessful alternative methods (data not shown), a dispersion of JAG21 was prepared using hydroxyethyl cellulose (HEC) and Tween 80. This new formulation method is described in the Materials and Methods. When this dispersion was imaged using a Nikon ECLIPSE E200 optical microscope set to 40x magnification, the average particle size of the JAG21 dispersion in HEC/Tween 80 was determined using an in-house image analysis program and was found to be 2.85 μm (Figure 7A). Material was re-sonciated the same way just prior to administration after being stored for 6 months and retained the same properties (Figure 7A) when imaged. Following administration of 2,000 highly virulent RH Strain tachyzoites intraperitoneally, the oral nanoformulation was administered by gavage using a 21 gauge needle. This was given either (1) as a single dose of 5, 10, or 20 mg/kg, or (2) three daily doses of 10 mg/kg given for the first 3 days after infection. After 5 days the RH strain tachyzoites in peritoneal fluid of each mouse were quantitated by measurement of YFP they expressed using a fluorimeter and by quantitating parasites present in peritoneal fluid using a hemocytometer. Parasite burden was reduced by ~60% 5 days later following the single doses of 10 and 20 mg/kg (representative experiment with 10 mg/kg shown in Figure 7B; *p* < 0.03) and markedly reduced with three doses of 10 mg/kg administered on each of the first 3 days after intraperitoneal injection of the virulent RH strain tachyzoites (Figure 7C, representative experiment, *p* < 0.001). This is the proof of principle that will facilitate media milling, dispersant, and a self disintegrating tablet in the future. JAG21 has real promise as a mature lead compound to treat both *T. gondii* and Plasmodium species infections.

## Discussion

*T. gondii* infections are highly prevalent and the impact of this disease can be devastatingly severe. Current treatments have toxicity or hypersensitivity side effects. New compounds that are without toxicity or hypersensitivity, and that are highly active against tachyzoites would be of considerable clinical usefulness. Further, no medicines are active against the encysted stage or definitively curative. In addition, malaria is lethal for 1 child every 11 s and a threat to travelers going to endemic areas. Development of drug resistance also increases the need for new anti-malarial compounds. Our goal in this work herein was to identify compounds highly effective against *T. gondii* and *P. falciparum*, and we believe we have achieved our goal by developing lead compounds with dual activity.

To further develop the THQ series, 54 compounds were synthesized to improve kinetic solubility, solubility in physiologically-relevant media (FaSSIF, FeSSIF), and metabolic stability (microsomes and hepatocytes), and other ADMET properties. Compounds JAG50 and JAG21 were identified as lead compounds, demonstrating potent inhibition on both tachyzoites and bradyzoites life stages and were not toxic to human cells in our *in vitro* model (HFF). In addition, both compounds displayed low nanomolar efficacy against multiple drug resistant strains of *P. falciparum in vitro*. JAG50 and JAG21 demonstrate promising ADMET properties, with JAG21 slightly superior due to the compound's longer metabolic stability in human and mouse microsomes.

A striking result with JAG21 in our *in vivo* parasite studies is the compound's high efficacy against *T. gondii* tachyzoites and bradyzoites. In our *P. berghei in viv*o model for malaria, we observed that a single dose causal prophylaxis in 5 C57BL/6 albino mice at 2.5 mpk dosed on day 0, 1 h after intravenous administration of 10,000 *P. berghei* sporozoites was achieved. Causal prophylaxis was also observed after a 3-dose treatment in 5 C57BL/6 albino mice at 0.6 mpk dosed on days −1, 0, and +1. A representative figure at a higher dose (5 mg/kg) is shown, and all experiments with the amounts mentioned above demonstrated identical high efficacy in luminescence, parasitemia, and survival results. This demonstrates that JAG21 functions better in this *in vivo* model than the ELQ 300 series where prodrug formulation is required to achieve solubility and efficacy, in contrast to the efficacy of JAG21 at 2.5 mg/kg in a single oral dose model resulting in cure without a prodrug. ELQ 300 (not the prodrug) was not effective at doses between 1 and 20 mg/kg although the prodrug was more effective (Doggett et al., 2012; Frueh et al., 2017).

JAG50 and JAG21 are lead compounds, with JAG21 being a superior compound due to its favorable predicted ADMET properties, potency, efficacy, and lack of toxicity. JAG21 demonstrates increased solubility and potential for advanced formulation. There also is potential for improving solubility and reducing toxicity further because of the larger binding pocket in the apicomplexan Cytbc1 enzyme compared with the mammalian Cytbc1 enzyme. This was determined by modeling occupancy of the structure, enzyme assays and empirically. We have created and tested additional compounds that take advantage of these properties, although none at present, have the proven ADMET and marked *in vivo* efficacy we found to be advantageous in our proof of principle studies of JAG21. At present, however, our mature lead compound has sufficient drug like properties to move to advanced formulations, suggesting increased bulk will not be needed to reduce toxicity. It has selectivity as demonstrated by our enzymatic, binding, and structure studies, although there are additional compounds that show even greater selectivity. It is highly effective in an oral nano preparation against *P. berghei's* three life cycle stages, and with early treatment appears to be capable of curing toxoplasmosis in immunocompetent mice. This work demonstrates the promising nature of this novel tetrahydroquinolone scaffold and mature lead compound. JAG21 has the potential to become an orally administered medicine or with partners, part of a medicine combination that is curative for toxoplasmosis and is a single dose cure for malaria. It is suitable for partnering with other compounds to obviate problems with selection of resistant mutants. We have demonstrated earlier that the parent compound with this new scaffold is synergistic with atovaquone and additive with cycloguanil (in proguanil) against *P. falciparum* (McPhillie et al., 2016). Herein, we also found synergy between JAG21 and atovaquone against *T. gondii* tachyzoites *in vitro*. This compound is a mature lead compound to treat both *T. gondii* and Plasmodium species infections. If utility and safety retained, and no toxicity appears in next stage studies, this compound may become suitable for treatment of *T. gondii* and *P. falciparum* infections.

## Conclusion

JAG21 has real promise as a mature lead compound to treat both *T. gondii* and Plasmodium species infections, demonstrated *in vitro* and *in vivo*. It has high efficacy against *T. gondii* tachyzoites and bradyzoites, and established encysted organisms. Treatment with a single low oral dose is effective for causal prophylaxis and radical cure *of P. berghei* infections. JAG 21 has complete efficacy against three life cycle stages of *P. berghei*. In terms of companion inhibitors, JAG21, a Q_i_ inhibitor, synergizes against tachyzoites with atovaquone (a Q_o_ inhibitor) *in vitro*. It appears able to contribute modestly to protection of immune compromised mice in conjunction with tafenoquine against an initially replicating, then G1 arrested, *T. gondii* parasite that shares key transcriptomic components with *P. cynomolgi* hypnozoites. Our mature lead compound has sufficient selectivity and drug-like properties to support ongoing efforts to further develop this compound through preparation of advanced formulations and testing in additional studies of pharmacokinetics, efficacy, and safety.

## Data Availability Statement

The EM map has been deposited at the EMDB (EMDB-11002). Structure coordinates are deposited and available from Protein Data Bank under the accession code: 6XVF.

## Ethics Statement

This animal study was reviewed by, approved by, and carried out in accordance with regulations of the University of Chicago IACUC and IBC and of The Home Office of the UK Government under the Animals [Scientific Procedures] Act 1986. All work in the UK with mice was covered by License PPL60/4568, Treatment and Prevention of Toxoplasmosis with approval by the University of Strathclyde ethical review board.

## Author Contributions

MM, RM, MH, HL, SNM, CF, CR, SPM, GB, SA, KR, RKP, and YZ conceptualized and designed the overall study. MM, RM, MH, and HL wrote manuscript. All authors wrote subparts of manuscript, performed experiments, and/or analyzed data. All authors reviewed and edited manuscript in final form.

## Conflict of Interest

RM, MM, CF, CR, KE, MH, QL, and HL are inventors on an International patent application PCT/US2016/067795 pertinent to the work in this study. RM has completed an unrelated literature review for Sanofi-Pasteur. The remaining authors declare that the research was conducted in the absence of any commercial or financial relationships that could be construed as a potential conflict of interest.
