# Supplementary material for: Potent Tetrahydroquinolone Eliminates Apicomplexan Parasites
Source: Front Cell Infect Microbiol. 2020 Jun 9;10:203. doi: 10.3389/fcimb.2020.00203 (PMC7311950; doi:10.3389/fcimb.2020.00203)

Supplemental : Potent tetrahydroquinolone eliminates apicomplexan parasites

Martin J. McPhillie[1,+] , Ying Zhou[2,+], Mark R. Hickman[3,+,++], James A. Gordon[1,++, +], Christopher R. Weber[4,+], Qigui Li[3], Patty J. Lee[3], Kangsa Amporndanai[5], Rachel M. Johnson[6,++], Heather Darby[1], Stuart Woods[7], Zhu-hong Li[8], Richard S. Priestley[9], Kurt D. Ristroph[10], Scott B. Biering[4, ++] , Kamal El Bissati[2], Seungmin Hwang[4++], Farida Esaa Hakim [2], Sarah M. Dovgin[2, ++] Joseph D. Lykins[2, ++], Lucy Roberts[7, ++], Kerrie Hargrave[7,++], Hua Cong[1,++], Anthony P. Sinai[11], Stephen P. Muench[6], Jitender P. Dubey[12] , Robert K. Prud'homme[10], Hernan A. Lorenzi[13+], Giancarlo A Biagini[9], Silvia N. Moreno[8.*] Craig W. Roberts[7], Svetlana V. Antonyuk[5,*], Colin W.G. Fishwick[1,*], Rima McLeod[2,14*]

[1] Organic Chemistry, School of Chemistry, The University of Leeds, Leeds, UK

[2] Department of Ophthalmology and Visual Sciences, The University of Chicago, Chicago, United States

[3]Experimental Therapeutics Branch, Walter Reed Army Institute of Research, United States

[4] Department of Pathology, The University of Chicago, Chicago, United States

[5] Department of Biochemistry and Systems Biology, Institute of Systems, Molecular and Integrative Biology, Faculty of Health and Life Sciences, The University of Liverpool, Liverpool, UK

[6] School of Biomedical Sciences, Faculty of Biological Sciences, and Astbury Centre for Structural Molecular Biology, The University of Leeds, Leeds, UK

[7]Strathclyde Institute of Pharmacy and Biomedical Sciences, The University of Strathclyde, Glasgow Scotland, UK

[8] Department of Cellular Biology, Center for Tropical and Emerging Global Diseases, Athens, GA, United States.

[9]Department of Tropical Disease Biology, The Liverpool School of Tropical Medicine, Liverpool, UK

[10] Department of Chemical and Biological Engineering, Princeton University, Princeton, New Jersey, United States

[11] Microbiology, Immunology and Molecular Genetics,The University of Kentucky College of Medicine, Lexington, United States

[12] Animal Parasitic Diseases Laboratory, USDA, ARS, Beltsville, Maryland, United States

[13] Department of Infectious Diseases,J.Craig Venter Institute, Rockville, Maryland, United States

[14] Department of Pediatrics (Infectious Diseases), Institute of Genomics, Genetics, and Systems Biology, Global Health Center, Toxoplasmosis Center, CHeSS, The College, University of Chicago, Chicago, IL, USA

[+]These authors contributed equally to this work

[*]To whom Correspondence should be addressed

[++]Currently at : Charles River, Chesterfield Park, Cambridge UK (JAG); Fort Detrick, Frederick, MD, USA (MRH); University of California at Berkeley, Berkeley, CA, USA (SBB); Monash University, Melbourne Australia (RMJ); VIR Biotechnology, San Francisco, CA,USA, (SH); Case Western Reserve University, Cleveland, OH, USA (SMD); Virginia Commonwealth University Health System, ,Richmond,VA, USA (JDL); Shandong University, Shandong, China(HC); University of Glasgow, Glasgow, Scotland (KH)

Supplement Part 1,
Part 2 is the excel sheet showing results of RPS13delta in human primary brain neuronal stem cells

<u>Index to supplemental materials:</u>
Additional detailed text for the section about "Companion Compounds, G1 Arrest, Persisters.Stasis, and a summary overview

Supplemental Table S1 (For fig 4, Data Collection Statistics)
Supplemental Table S2 (Table to accompany Fig 6)

Additional detailed text for the section about Companion Compounds, G1 Arrest and Persisters and brief Summary

Companion Compounds, G1Arrest and Persisters (more detailed text)
Similar *RPS 13Δ* and *Plasmodium* Hypnozoites' Transcriptomes
Experiments testing the compounds against the EGS strain had some surprising findings. While true cysts *in vitro* appeared to be completely eliminated by treatment, or their number significantly reduced, a very small number of parasites did appear to persist in tight, clustered, cyst-like structures, or pseudo-cysts, and there appeared to be small punctate life forms that resembled very small tachyzoites. One possible explanation is that the dolichos-staining organisms that remain 48 hours after treatment are in a separate, hypnozoite-like G1 arrested life stage that is not affected by the compounds. Hypnozoites are a practical problem for attempts to definitively cure and treat malaria, especially *P.vivax* and *P.ovale,* and also *P. falciparum.* Further, to reduce the possibility of resistance developing especially for *Plasmodia*, a companion drug that can work additively or synergistically with our compounds with this new scaffold may be useful. Primaquine and tafenoquine, which are the only medicines known to treat the hypnozoite stage of *Plasmodium vivax* and *P. ovale,* may be potential candidates as shown with our G1 arrested organism. Further, we also had demonstrated synergy of an earlier generation related compound with atovaquone, and additive effect with cycloguanil for *P. falciparum*. Herein, we find a similar synergistic effect of atovaquone with JAG21. JAG21, which is a cytochrome *bc* Qi inhibitor, synergizes with the Qo inhibitor atovaquone but not pyrimethamine. It will be of interest in future work to determine whether synergy with other compounds that target unique mitochondrial enzymes, other domains of cytochrome b/c, or other molecular targets such as an inhibitor of calcium kinase.

Primaquine is routinely used and tafenoquine has now been evaluated (St. Jean al 2016; Lacerda et al 2019; Llanos-Cuentas et al 2019) and recently FDA approved for use in the treatment of residual hypnozoites capable of recrudescence in human *P. vivax*, *ovale* and to a lesser extent *falciparum* malarias, and modeled in the non-human primate *P. cynomolgi* malaria. These hypnozoites are resistant to all other known medicines and require the presence of mature hepatocytes with CYP2D6 enzymes to metabolize the primaquine or tafenoquine into a reactive intermediate that has a toxic reactive electron that kills the hypnozoite. Thus, these aminoquinolones are used in conjunction with drugs that treat the active blood stages of *Plasmodia* infection (Lacerda et al , 2019; Llanos-Cuentas et al, 2019). Malarone, a combination of atovaquone that targets cytochrome *b,* and proguanil, is not effective against hypnozoites(Llanos-Cuentas et al, 2019). There are published transcriptomes of malaria hypnozoites obtained by laser capture from livers from humanized mice reconstituted with human red blood cells and mature hepatocytes which can metabolize tafenoquine and primaquine. These hypnozoite transcriptomes were obtained with single cell RNA sequencing and laser capture (Cubi et al 2017). The potential for recrudescence of the malaria hypnozoite was reminiscent of a *Toxoplasma* parasite we had

created earlier.

JAG21 Plus Tafenoquine

Because of these similarities we performed the following experiment in interferon γ receptor knockout mice to determine whether tafenoquine and JAG21 might act together against dormant (tafenoquine) and residual active parasites (JAG21) present in the initial challenge inoculum. Tafenoquine and JAG21 were used alone or together to treat the G1 arrested parasite and any residual active parasites present early in the infection, in the same way tafenoquine is added to malaria blood stage active drugs such as chloroquine to treat hypnozoite and blood stage malaria[39]. This was done knowing that for a short time after infection, without tetracycline present, that there would be some replicating Δ*RPS13* (Hutson et al, 2010*)* completing the cell cycle if they already had passed through G1. Thus we could determine whether tafenoquine or JAG21 or the two together might contribute to improved later survival by contributing to inhibition or killing of some G1 arrested organisms. *S*ubsequent treatment with tetracycline was administered to determine whether there was an effect on relapse with administration of tetracycline on the conditional knockout RPS13Δ in these immunodeficient mice. The combination of JAG21 and tafenoquine had a modest, early effect together on transiently improving survival when tetracycline was added when compared with JAG21 or tafenoquine alone. Up to the day when mice that received JAG21 and Tafenoquine began to die the combination appeared to be protective (Number of mice alive at time before combined treatment mice died: Initial experiment 9/15[controls receiving diluent, or either compound alone] vs 0/5 [Jag21+Taf] (p=0.03); Replicate run, 6/20 vs 0/9(p=0.08); Grouped replicate experiments 15/35 vs 0/14[p=0.002])(**Fig. 6C.S2**). These results were based on this time sensitive hypothesis that prolonged survival could be due to differently vulnerable parasite populations. P values are one sided**.**

Optimization of formulation will be needed to more fully and definitively test this hypothesis. Tetracycline was added to turn the *RPS13* gene on and convert the organism to a virulent parasite. Tafenoquine only has an effect in preventing relapse in malaria in the presence of well differentiated hepatic cells, which metabolize tafenoquine to a toxic charged molecule which destroys hypnozoites *in vivo* and when used in conjunction with drugs such as chloroquine that also treat the blood stage. It has no effect by itself. For immune compromised persons, if there is a *Toxoplasma* parasite comparable to this G1 arrested parasite that contributes to illness, this work raises the possibility that with further optimization of dose that this combination of compounds could be found to be useful. Perhaps this *RPS13Δ* organism also might prove useful in the future as a model of a G1 arrested apicomplexan parasite amenable for *in vitro* compound screening in a way pertinent to discovery of other inhibitors of malaria hypnozoites, if the similarity in transcriptomes truly reflects, and is further proven to be, a similar mechanism for dormancy of the malaria hypnozoite and Δ*RPS13*.

Summary

In summary, herein, our modifications optimized ADMET and resulted in a compound, JAG21, that is potent against *T. gondii* tachyzoites (IC_{90} <50 nM), bradyzoites (IC_{90} <1 μM), and drug resistant *Plasmodium falciparum in vitro* (IC_{90} <50 nM) with no detected toxicity to human HepG2 cells (IC_{50} >17 μM) or HFF (IC_{50} >7.5 μM). Further, we found that this compound demonstrates metabolic stability in assays against human and mouse liver microsomal activity and improved aqueous solubility at pH 7.4. This compound displays a balanced set of physicochemical and pharmacologic properties, including no inhibition of hERG or CYP enzymes, a long (days in

humans), predicted half-life and a predicted ability to cross the blood brain barrier. *In vivo, Toxoplasma* tachyzoites were cleared from mice at a dose of 5 mg/kg/day (IP) with no cysts found in treated mice at 30 days after discontinuing treatment. We found that JAG21 significantly, markedly reduces established cysts *in vivo* (p=0.03) when treatment is initiated 30 days after infection. JAG21 acted in conjunction with tafenoquine (3mg/kg single dose) to modestly and partially protect against a G1 arrested *T. gondii* parasite that could persist in interferon ϒ knockout mice, which was reminiscent of a similar effect of tafenoquine on malaria hypnozoites, which share a similar transcriptome demonstrating G1 arrest. Causal prophylaxis and radical cure was achieved after *P. berghei* sporozoite infection with three days oral administration of a nanoformulation of JAG 21 in HECT at a dose of 0.625mg/kg, and with a single dose of 2.5mg/kg. There was no *Plasmodia* parasitemia observed and 100% survival at 30 days post infection. This mature lead compound has improved solubility and diminished toxicity relative to other cytochrome *b* Qi inhibitors, without formulation as a pro-drug. Selectivity for the apicomplexan enzyme relative to mammalian enzymes was demonstrated with co-crystallography, binding and enzyme assays. JAG21 combined with tafenoquine also very slightly prolongs survival in immune compromised mice infected with a conditional G1 arrested tachyzoite form of *T. gondii* when the G1 arrest is rescued. This G1 arrested *T. gondii* organism resembles the malaria hypnozoite transcriptionally and modulates similar pathways. Moreover, without ATc the transcriptome of *T. gondii* Δ*RPS13* is compatible with a parasite transitioning from an active replicating form to a dormant stage, reflected by the downregulation of genes typical of the S and M stages of the cell cycle, and of genes that participate in energy metabolism and virulence (**Fig. 6B, D** and **Supplemental Table 2**). Further improvements in solubility and an even better therapeutic index may be possible in the future by taking advantage of new substituents defined by SAR and enhanced selectivity due to the larger binding pocket in the parasite enzyme relative to the mammalian enzyme, or achieved through further advances in formulation. Herein we identify a mature lead compound that is highly efficacious against *T. gondii* tachyzoites and bradyzoites *in vitro* and *in vivo*, *in vitro* against an array of drug resistant strains of *P. falciparum,* and *in vivo* against *P. berghei* infections' three life cycle stages. In 100% of mice after administration of JAG21 in a single oral dose of 2.5 mg/kg or after three days of oral dosing at 0.625 mg/kg there was causal prophylaxis and radical cure, and a formulation was created that is stable and effective orally with three doses against highly virulent *Toxoplasma* tachyzoites. This is the proof of principle that will facilitate media milling , dispersants and a self disintegrating tablet in the future. JAG21 has real promise as a mature lead compound to treat both *T.gondii* and Plasmodium spp. infection

Table S1. Data collection and refinement statistics for bc1-JAG021.

	***bc$_1$*-JAG021**
**Data collection**	
Space group	$P6_522$
Cell dimensions	
a, b, c (Å)	209.87, 209.87, 342.46
α, β, γ (°)	90°, 90°, 120°
Resolution (Å)	90.88-3.45 (3.56-3.45)
R_{merge} (%)	22.5 (111.0)
R_{pim} (%)	6.8 (33.4)
I/σI	8.6 (2.4)
Completeness (%)	91.4 (92.8)
Redundancy	11.4 (11.6)
Wilson B-factor (Å^2)	87.4
**Refinement**	
Resolution (Å)	90.88-3.45
No. reflection	48,681
R_{work}/R_{free}	21.74/23.24
No. atoms	
Protein	15,505
Inhibitor	30
Water	19
Other ligands	567
B-factors	
Protein	164.73
Inhibitor	143.91
Water	59.81
Other ligands	180.69
R.m.s. deviations	
Bond length (Å)	0.008
Bond angle (°)	1.426
PDB code	6XVF

* data in brackets are for the last shell

Table S1B. Data collection statistics for the cryoEM reconstruction of cytochrome bc1.

	bc*₁*-JAG
**Detector**	Falcon III
**Detector mode**	Integrating
**Voltage (kV)**	300
**Pixel size (Å)**	1.065
**Defocus (µm)**	-1 to -3.5
**Total dose (e^-/Å)**	66
**No. of frames**	59
**Exposure time (s)**	1.5
**Dose per frame**	1.12
**No. of micrographs**	5,356
**Total particle No.**	439,009
**Final particle No.**	211,916
**Resolution**	3.3 Å

Figure S1. JAG21 reduced immunostained material with or without Tafenoquine. Results were similar when cysts were quantitated microscopically.This shows pooled results of two replicate trials

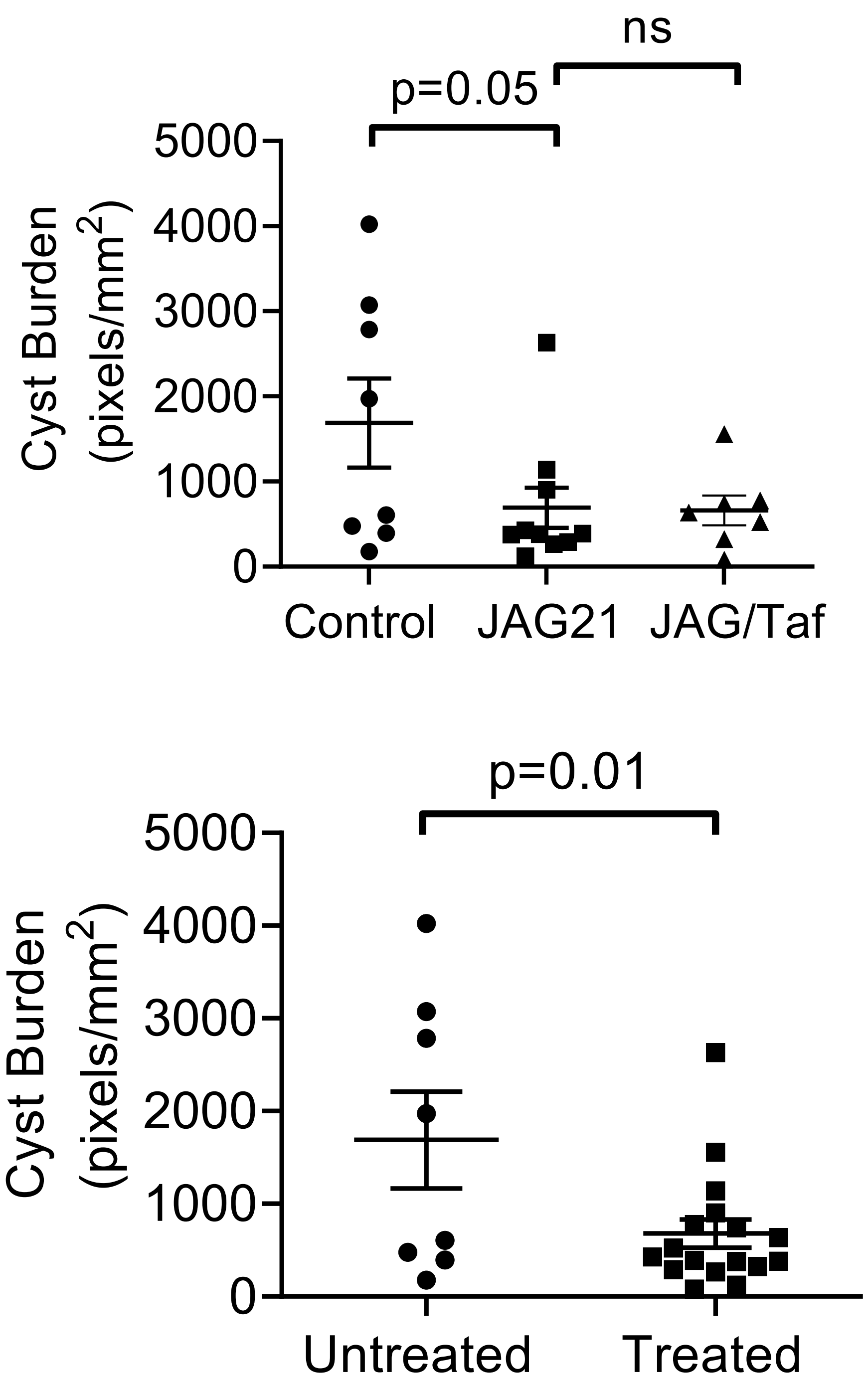

Figure S2. RPS13Δ in IFNγ receptor knockout mice. RPS13Δ in IFNγ receptor knockout mice created amorphous immunoperoxidase stained material in spleen and liver 7 and 14 days after infection when no tetracycline was given. The brown material looked less recognizable as *T.gondii* at 14, than at 7 days after initiation of infection**. A**. 7 days and 14 days after infection**. B.** When aTet is given. When a Tet was given to these mice in their drinking water after 7 or 14 days, parasites replicated and were lethal for ~50-80% of the mice**. C , D**. Design of experiment with treatment and primary data and Kaplan Meier analysis for this experiment. is shown When similarly infected mice were first treated on day -1 with tafenoquine, and for the first 14 days with JAG21 with each compound alone or the two together we found that the combination of JAG21 and tafenoquine prolonged survival modestly, although ultimately mice in that group also died (C,D). The pale yellow beige shows initial enhanced survival suggesting that the combination was modestly better than each alone, as shown in **Fig.6C**.. A, B, demonstrates that G1 arrested organisms can persist in immune incompetent mice and although even with immunoperoxidase staining, organisms may not be easily identified morphologically and can recrudesce. C,D suggested that dosing in those with immune compromise may need to be optimized and possibly continued for the duration of the immune compromise.**E.** Immunostaining after aTet given in a tafenoquine treated mouse demonstrated multiple single organisms at time of death of the mouse**.** C,D,**E**, demonstrate that adding tafenoquine to JAG21 prolongs survival modestly but as dosed did not result in durable protection after .

A

INFγ KO infected with RH-RPS13Δ, without a-tet

D7
Spleen
Stained
with anti-
T. gondii
antigen
antibody

D14

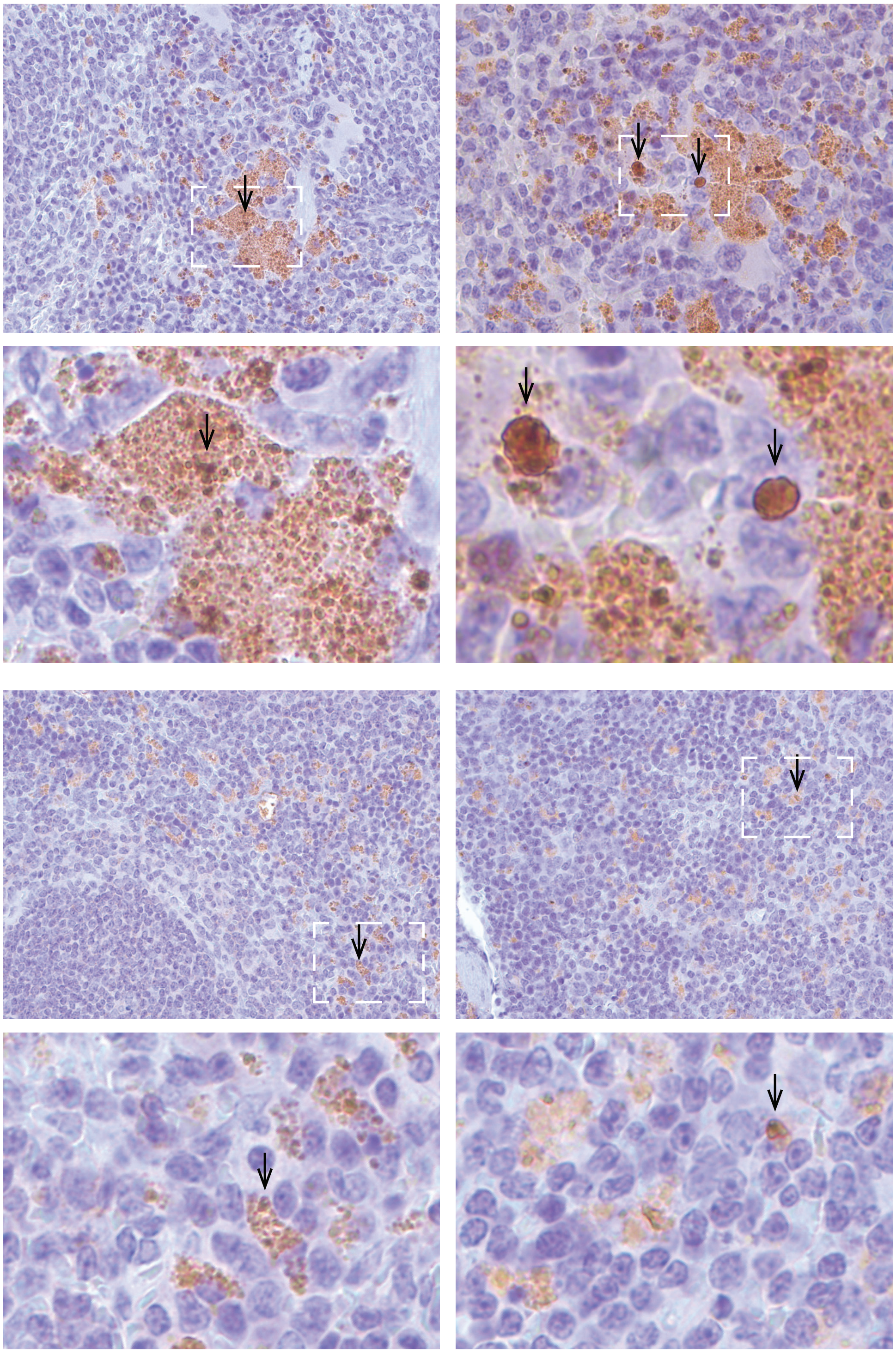

B Rescued with aTet after 14 days: INFγ KO mouse infected with RPS13Δ, with tet from d7, mouse was euthanized when it became too sick

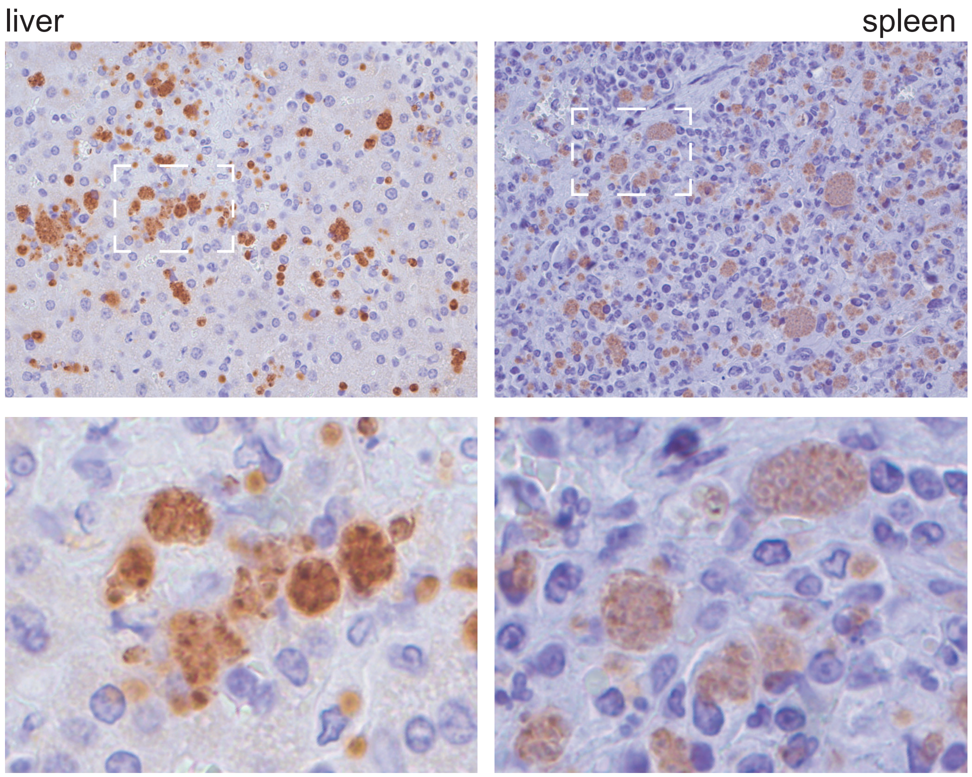

C, D

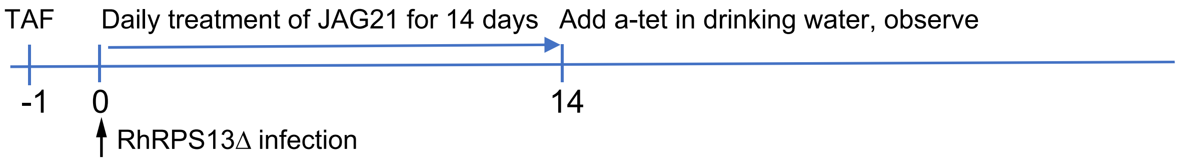

Experiment 1				
total number of mice to start	5	5	5	6
day of death	DMSO	Tafenoquine	JAG21	Tafenoquine +JAG21
10		1		
12			1	
13	1	1		
15		1		
18	1		1	
24		1		
28			1	
36				1
41				1
number of mice left at 42	3	1	2	4
Experiment 2				
total number of mice to start	10	5	5	9
day of death	DMSO	Tafenoquine	JAG21	Tafenoquine +JAG21
12		1		
13	1			
15	1			
17	1			
19		1		
20			1	
21				1
22			1	2
23	1			3
25		1		
27				2
number of mice left at 37	6	2	3	1

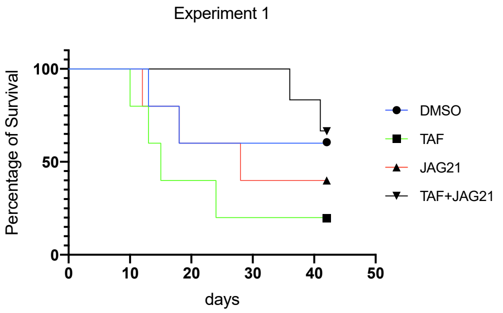

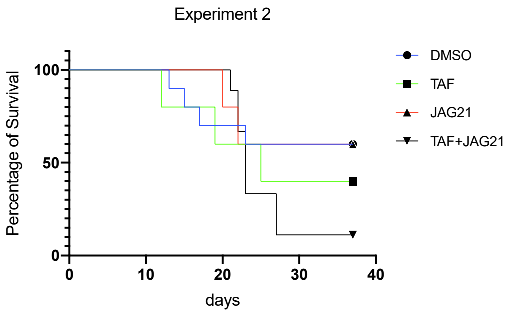

E

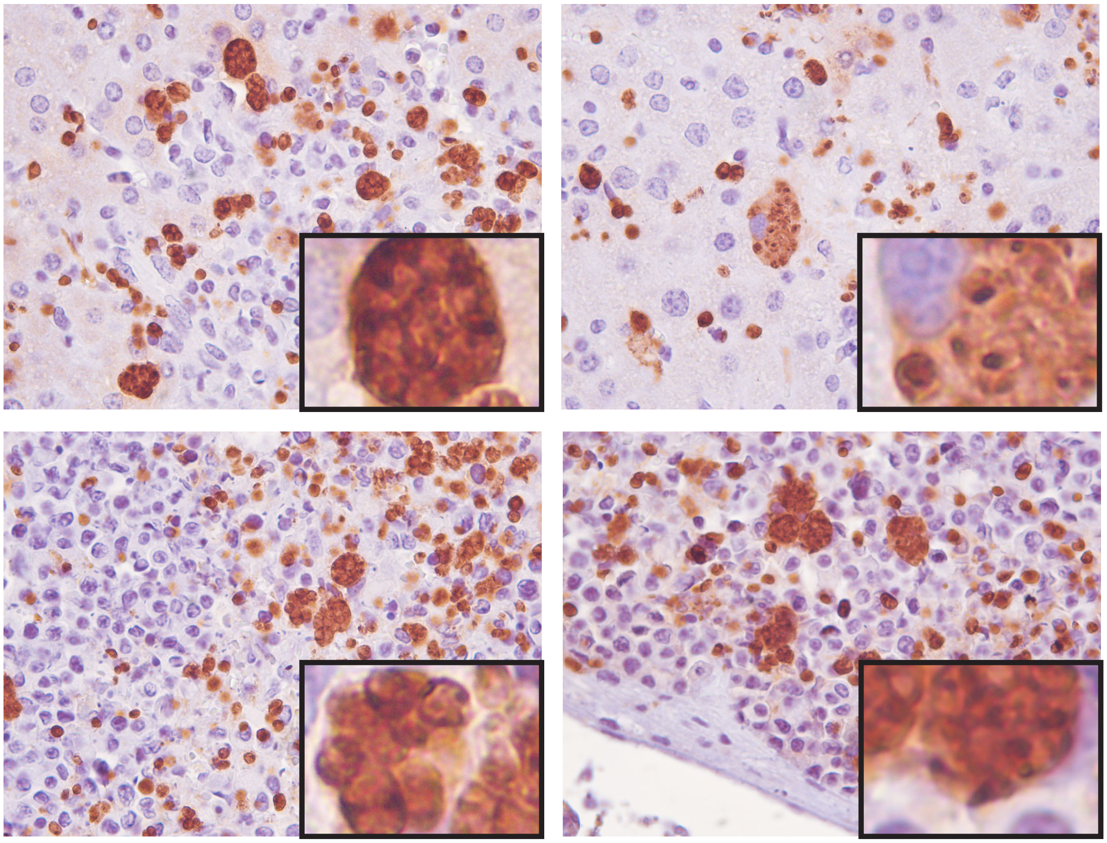

Supplement: Supplementary file 1 [file Data_Sheet_1.pdf]
